# A Novel Method for Motion Blur Detection and Quantification Using Signal Analysis on a Controlled Empirical Image Dataset

**DOI:** 10.3390/s26082360

**Published:** 2026-04-11

**Authors:** Woottichai Nonsakhoo, Saiyan Saiyod

**Affiliations:** Hardware-Human Interface and Communications Laboratory (H2I-Comm Lab), Department of Computer Science, College of Computing, Khon Kaen University, Khon Kaen 40002, Thailand; nonsakhoo@cassia.kku.ac.th

**Keywords:** motion blur, motion blur quantification, scanline analysis, self-similarity, movement artifact, reference origin estimation, velocity estimation, polynomial regression, controlled empirical dataset

## Abstract

Motion blur degrades single-frame imaging when relative motion occurs during sensor exposure; yet, quantitative validation is difficult because ground-truth motion parameters are rarely available in real images. This paper presents an interpretable, measure-first framework for detecting, localizing, and quantifying motion blur in single-frame grayscale images under a validated operating condition of one-dimensional horizontal uniform motion. The method analyzes each image row as a one-dimensional spatial signal, where Movement Artifact denotes the scanline-level imprint of motion blur retained in the legacy algorithm names MAPE and MAQ. The pipeline combines three stages: Movement Artifact Position Estimation (MAPE) using scanline self-similarity, Reference Origin Point Estimation (ROPE) using robust structural trends, and Movement Artifact Quantification (MAQ), which summarizes blur magnitude as an average horizontal spatial displacement after adaptive filtering. The pipeline is evaluated on a controlled empirical dataset of 110 images of a high-contrast marker acquired at known tangential velocities from 0.0 to 1.0 m/s in 0.1 m/s increments (10 images per level). MAPE achieves 70–90% detection rates across velocities, and ROPE localizes reference origins with 97–99% detection. An empirical polynomial mapping from MAQ to velocity attains *R*^2^ = 0.9900 with RMSE 0.0229 m/s and MAE 0.0221 m/s over 0.0–0.7 m/s, enabling calibrated velocity estimates from blur measurements within the validated regime. An extended additive-noise robustness analysis further shows that severe perturbation can preserve candidate self-similarity responses while progressively destabilizing reference-origin localization and MAQ pairing, thereby clarifying the empirical boundary of the current controlled single-marker regime. The approach is not claimed to generalize to uncontrolled scenes, non-uniform blur, or multi-dimensional and non-rigid motion.

## 1. Introduction

Imaging underpins many modern decision pipelines, ranging from human interpretation to automated analysis and quality assurance. In such settings, estimating degradation parameters can be as valuable as restoration itself, enabling quality gating, adaptive parameter selection, and targeted compensation.

Motion blur is a common degradation in single-frame imaging when relative motion occurs during sensor exposure. Quantifying motion blur is difficult because ground-truth motion parameters are typically unavailable in real images, making validation and calibration challenging. As a single-frame inverse problem, different motion trajectories can yield similar observations, and many approaches are therefore assessed indirectly (e.g., via restoration quality) rather than against known motion parameters [1,2].

This work was originally motivated by an ultrasound-screening pipeline context in which automated analysis is highly sensitive to acquisition quality and motion-induced distortions. Related efforts include image enhancement, automated analysis, and coherence/correlation-based processing pipelines [3,4,5,6,7,8]; more broadly, clinically oriented deep learning systems and surveys highlight both the opportunities and the sensitivity of automated decision pipelines to data quality [9,10]. Motion corruption and compensation have also been studied across modalities and acquisition settings, including motion estimation/correction in medical imaging [11,12,13,14] and supporting signal-processing components [15,16,17]. While these references provide historical motivation and system context, the technical contribution of this manuscript is a domain-agnostic, single-frame motion blur quantification pipeline.

Appendix A summarizes the historical medical-imaging motivation and system context; the remainder of this manuscript is written to be domain-agnostic.

Prior work on motion blur spans multiple directions. Motion blur is related to, but distinct from, generic noise models that are often handled by classical denoising. Standard spatial and multiresolution filtering methods are effective for many forms of additive noise [18,19,20], and non-local strategies have proven powerful for structure-preserving denoising [21]. In contrast, exposure-time integration under motion produces a structured, content-dependent distortion; blur-aware modeling and estimation are therefore required. Related formulations also arise in settings that combine blur with multiplicative noise and speckle components [22,23,24,25,26].

This includes motion-artifact compensation pipelines [27,28,29,30,31,32,33,34,35,36] and single-image deblurring/blur estimation, including uniform camera shake models [37], coded-aperture and parametric blur estimation [38,39,40], and non-uniform blur modeling [41,42]. Evaluation remains subtle: even when restoration quality appears strong, blind deconvolution objectives can admit degenerate explanations, and results may depend strongly on priors and protocol details [2]. Surveys summarize both traditional and deep approaches and highlight persistent challenges [43,44].

Frequency-domain analysis can provide useful intuition about how motion redistributes energy across spatial frequencies, and fast implementations make these tools practical [45]. Phase- and correlation-based signatures in the frequency domain can also carry motion-pattern information [46], and correlation/self-similarity cues have been used for blur parameter estimation and motion analysis [47,48,49]. These spatial and spectral viewpoints are complementary descriptions of the same structured blur phenomenon. In the present study, we retain the scanline domain operationally because it preserves spatial interpretability and supports direct calibration of a displacement quantity against known motion labels. Recent optimization-based and learning-based deblurring methods demonstrate strong restoration performance on complex blur [50,51,52,53,54,55,56], but restoration quality does not necessarily provide an interpretable estimate of blur magnitude; learning-based approaches also often depend on large training sets and paired data, motivating alternatives such as unpaired training [57]. More broadly, deep learning is widely used for inverse problems in imaging [58,59,60,61], but interpretability and validation against known physical parameters remain recurring concerns.

Quantitative metrics are essential for comparing blur-affected images and for linking algorithm outputs to measurable degradation. Widely used image quality measures and their extensions provide principled evaluation tools [62,63,64,65]. Benchmarking efforts also emphasize that results derived from purely synthetic data may not transfer reliably to empirically captured data, motivating carefully controlled datasets and reproducible evaluation protocols [66].

Despite this breadth, relatively few end-to-end methods produce spatially localized, interpretable blur measurements that can be directly validated against known motion parameters in empirical data.

To address this gap, we construct a controlled empirical single-frame image dataset in which object velocity is known at capture time and used as ground truth. The dataset spans tangential velocities from 0.0 to 1.0m/s in 0.1m/s increments with multiple captures per level. Velocity labels are obtained from the controlled motion mechanism used during capture, and repeated captures per level help mitigate run-to-run acquisition variability. This controlled design supports quantitative evaluation and an empirical mapping from MAQ displacement to velocity under a validated operating condition.

The scope of this paper is deliberately constrained to the validated operating condition of *one-dimensional horizontal uniform motion blur*. The framework does not claim performance on uncontrolled real-world scenes, multi-dimensional/non-rigid motion, or domain-specific imaging data where ground-truth motion labels are unavailable.

This work presents a signal-analysis framework for detecting, localizing, and quantifying motion blur in single-frame empirical images under this controlled, ground-truth-validatable setting. The approach interprets each image row as a one-dimensional spatial signal and extracts interpretable measurements through three algorithms: Movement Artifact Position Estimation (MAPE), Reference Origin Point Estimation (ROPE), and Movement Artifact Quantification (MAQ). Operationally, the measured quantity is the horizontal displacement imprinted along each scanline by the validated drift term of one-dimensional horizontal motion. We use *motion blur* throughout; *Movement Artifact* appears only in the historical algorithm names (MAPE/MAQ). The proposed pipeline produces scanline-domain measurements and does not require operational Fourier-domain computation; frequency-domain discussion is included only as contextual intuition.

In summary, this paper makes three main contributions:

- A measure-first signal-analysis pipeline (MAPE–ROPE–MAQ) that detects likely blur-affected scanlines, estimates reference origin points, and quantifies blur magnitude as an interpretable spatial displacement.
- A controlled empirical single-frame image dataset with known tangential velocities at capture time, enabling quantitative, ground-truth validation of motion-blur quantification.
- An empirical mapping from MAQ displacement to velocity under the validated assumption of one-dimensional horizontal uniform motion blur.

The present manuscript should, therefore, be read as the first phase of a staged program focused on blur magnitude under controlled one-dimensional motion; a subsequent controlled extension to blur direction estimation has been investigated separately [67].

The remainder of this paper is organized as follows. Section 2 provides the proposed methodology for motion blur detection and quantification. Section 3 details the experimental setup, and Section 4 presents results and analysis. Section 5 discusses implications, limitations, and future directions, and Section 6 concludes the paper.

## 2. Proposed Motion Blur Detection and Quantification Methodology

**Figure 1:**
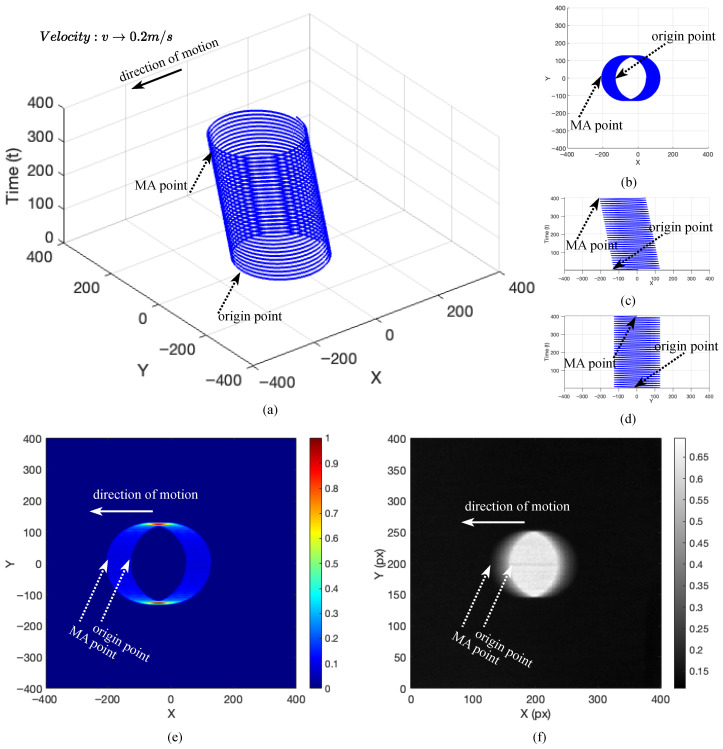
Illustrative depiction of motion-blur formation. Subfigures show: (**a**) three-dimensional helical trajectory in (x, y, t) space, (**b**) top-down x–y projection of the trajectory path, (**c**) x–time evolution showing horizontal drift superimposed on oscillatory motion, (**d**) y–time evolution showing vertical oscillation, (**e**) kernel-based 2D spatial projection mimicking sensor exposure accumulation, and (**f**) corresponding empirical high-contrast marker image for reference. The helical trajectory is used only to visualize exposure-time accumulation and the resulting spatial smear; the validated MAPE–ROPE–MAQ pipeline measures the horizontal scanline displacement induced by the drift term -vt under one-dimensional horizontal uniform motion.

**Figure 2:**
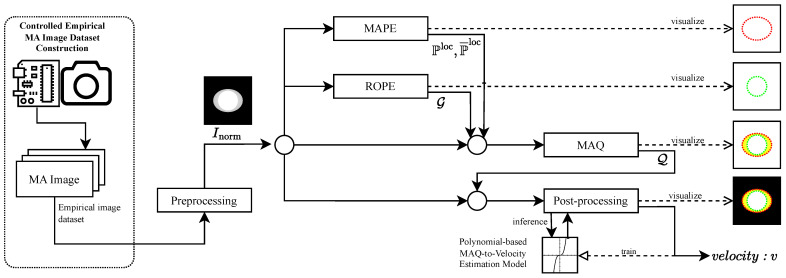
Proposed motion blur detection and quantification methodology overview.

The proposed methodology is presented as a sequential, scanline-domain pipeline for the detection and quantification of motion blur in grayscale images. Figure 1 provides an illustrative basis for motion-blur formation that motivates the signal-analysis perspective, and Figure 2 summarizes the end-to-end workflow. Within the validated operating condition, the operational quantity of interest is the horizontal scanline displacement induced by the drift term of the motion model. The illustrative construction in Figure 1 is included only to motivate why a one-dimensional scanline representation is informative. We begin with an illustrative signal representation that motivates the validated operating condition (Section 2.1), describe the controlled empirical dataset (Section 2.2) and preprocessing (Section 2.3), and then detail the three-stage measurement pipeline: MAPE for scanline self-similarity-based localization (Section 2.4), ROPE for reference origin estimation (Section 2.5), and MAQ for displacement quantification (Section 2.6). Finally, post-processing maps MAQ measurements to velocity estimates via polynomial regression (Section 2.7).

### 2.1. Mathematical Signal Representation of Motion-Blur Properties (Illustrative)

This subsection provides mathematical intuition and an illustrative simulation of motion-blur formation using complex exponentials. It is included to motivate the modeling perspective and to provide a transition to the proposed pipeline; it is not an operational step used by the detection and quantification algorithms, which operate directly on one-dimensional scanline signals extracted from empirical images. In particular, the validated algorithms do not attempt to recover a full three-dimensional trajectory from a single frame; they use this illustration only to motivate why the horizontal drift imprint can be measured from one-dimensional scanlines.

To illustrate motion blur formation, we consider a simple controlled model: a high-contrast point moves at constant velocity across a uniform background during exposure. The illustration is grounded in complex exponentials, starting with Euler’s identity:

(1) $$e^{i\pi }+1=0$$

which unifies *e*, *i*, and π, and reveals the link between exponential, rotational, and circular structures. More generally, Euler’s formula $e^{ix}=\cos x+i\sin x$ shows how complex exponentials encode rotation in the plane, with (cosx,sinx) tracing a circle as *x* varies [68]. The real and imaginary parts correspond to orthogonal sinusoidal oscillations, and *x* represents phase or angular position. This formulation gains more expressiveness when cast in polar coordinates:

(2) $$e^{i\theta }=r\left(\cos\theta +i\sin\theta \right)$$

where r=1 on the unit circle. In this form, we emphasize the rotating nature of the complex exponential, making it ideal for encoding cyclical motion, oscillations, and frequency content in spatial and temporal signals. To formalize this behavior analytically, we recall the real and imaginary decompositions:

(3) $$\begin{array}{cc} \cos x & =ℜ\left(e^{ix}\right)=\frac{e^{ix}+e^{-ix}}{2} \end{array}$$

(4) $$\begin{array}{cc} \sin x & =ℑ\left(e^{ix}\right)=\frac{e^{ix}-e^{-ix}}{2i} \end{array}$$

In the remainder of the paper, the processing pipeline does not require explicit Fourier-domain computation; instead, it relies on scanline-wise signal structure (self-similarity, slope trends, and robust pairing) to detect and quantify motion blur in empirical images.

To bridge this intuition to the formal signal representation, we adopt a simple exposure-like view: the motion trajectory is sampled at discrete times $t_{n}=ns$, n=0,…,N−1, and each sample contributes locally to a 2D intensity map via accumulation with a compact spatial kernel *K* (a discrete Gaussian kernel for smooth, sub-pixel rendering in Figure 1e). This accumulation is an intuitive proxy for integration over exposure time, producing a spatial “smear” whose extent grows with the drift term −vt under the validated one-dimensional horizontal uniform motion assumption. The operational pipeline does not require this simulation; rather, it motivates why one-dimensional scanlines can contain repeated/self-similar structure at displacements that relate to motion blur magnitude.

For interpretability, we describe a simple motion-induced distortion model using complex exponentials and their real-imaginary decompositions. Specifically, the continuous signal representation

(5) $$e^{i\omega t}=\cos\left(\omega t\right)+i\sin\left(\omega t\right)$$

serves as the basis for encoding rotational and oscillatory motion. To incorporate translational movement, the parametric trajectory is formulated by combining the oscillatory terms with a linear drift component, yielding the following discrete simulation model:

(6) $$X\left(t\right)=\left(A\cos(\omega t)-vt,\;A\sin(\omega t),\;t\right)$$

where *A* is the amplitude, ω is the angular frequency, *v* is the velocity, and *t* is the temporal index. This formulation provides a mathematically consistent transition from the theoretical signal model to the practical simulation of motion blur, capturing both the cyclical and translational dynamics that underlie blur formation. This parametric representation can also be expressed in the following compact complex exponential form:

(7) $$X\left(t\right)=Ae^{i\omega t}-vt$$

which highlights the underlying rotational and translational dynamics in a unified mathematical framework. Appendix B yields the corresponding equivalence between real-valued and complex signal representations. This representation can be interpreted component-wise. The *x*-component, Acos(ωt)−vt, superimposes an oscillatory term with a constant-velocity drift; for v>0, the trajectory translates leftward over time, consistent with horizontal smearing under the validated one-dimensional motion assumption. The *y*-component, Asin(ωt), provides the orthogonal (quadrature) oscillation associated with the complex exponential. The third component, *t*, indexes time and yields a helical trajectory in (x,y,t) space. Accordingly, within the controlled empirical setting, the operational target of MAPE–ROPE–MAQ is the scanline-wise horizontal drift imprint rather than the rotational component itself. Figure 1 summarizes this illustration: (a) Three-dimensional trajectory of the simulated motion signal; (b) top-down 2D projection; (c) *x*–time plot; (d) *y*–time plot; (e) kernel-based sub-pixel rendering; and (f) a corresponding empirical image example (inverted for visualization).

The mathematical modeling result is a spatially warped spiral whose appearance is governed by motion parameters (e.g., *v*, *A*, ω) and sampling. In this illustrative view, motion blur couples scene structure with the motion trajectory during exposure, yielding structured, content-dependent distortions rather than a simple additive-noise effect. By comparison, a common mixed-noise observation model in digital signal processing can be written as

(8) $$y\left(t\right)=s\left(t\right)\;n_{\mathrm{mult}}\left(t\right)+n_{\mathrm{add}}\left(t\right)$$

where s(t) is the underlying signal, $n_{\mathrm{mult}}\left(t\right)$ represents multiplicative corruption (e.g., gain fluctuations or speckle-like effects), and $n_{\mathrm{add}}\left(t\right)$ represents additive noise [18,19,25]. This paper does not estimate or remove these noise terms; the model is included only to contrast generic noise assumptions with exposure-time motion blur structure. The amplitude *A* controls the strength of the contribution, while *v* governs the extent of the drift that manifests as horizontal smearing under the validated one-dimensional motion assumption.

To translate this 3D parametric motion into a 2D image frame—akin to a single exposure from the camera—we discretize the parametric equation by sampling $t_{n}=ns$ for n=0,1,…,N−1, and define the resulting intensity projection I(x,y) as an accumulation over time, where each sampled point contributes a brightness signal at its location:

(9) $$\begin{array}{cc} I\left(x,y\right)=\sum_{n=0}^{N-1} & \delta \left(x-\left[A\cos\left(\omega t_{n}\right)-vt_{n}\right]\right)\\ \; & \cdot \;\;\delta \left(y-A\sin(\omega t_{n})\right) \end{array}$$

where δ(·) is the unit impulse function, representing an idealized point contribution in continuous space. In a discrete digital image domain, this becomes a 2D intensity array I[i,j], where each spatial sample is mapped to pixel indices via rounding:

$$x_{n}=A\cos\left(\omega t_{n}\right)-vt_{n},\;y_{n}=A\sin\left(\omega t_{n}\right)$$

(10) $$I\left[i,j\right]=\sum_{n=0}^{N-1}K\left(i-\mathrm{round}\left(x_{n}\right),j-\mathrm{round}\left(y_{n}\right)\right)$$

In this formulation, K(m,n) is a discrete kernel function that governs how the energy of each sample is distributed to nearby pixels. A simple choice is the discrete Kronecker delta:

(11) $$K\left(m,n\right)=\left\{\begin{array}{cc} 1, & \mathrm{if}\;m=0\;\mathrm{and}\;n=0\\ 0, & \mathrm{otherwise} \end{array}\right.$$

which places all energy at the closest integer coordinate (i.e., a pixel hit). For smoother rendering or sub-pixel simulation, a discrete Gaussian kernel centered at each sample is used:

(12) $$K\left(m,n\right)=\exp\left(-\frac{m^{2}+n^{2}}{2\sigma^{2}}\right),\;\sigma >0$$

where σ controls the spread of the kernel, allowing for a more gradual intensity falloff around each sample point. Appendix C provides a detailed explanation of how the 3D trajectory is projected onto a 2D image. This approach allows image blurring to emerge naturally from interpolation and motion aliasing, improving visual realism in high-resolution simulations. The complete 2D image I[i,j] thus serves as the modeled output of a camera frame capturing the dot’s motion, where spatial deformation encodes velocity-induced artifact behavior.

This mathematical modeling framework provides a rigorous foundation for simulating and visualizing motion-blur formation in digital images. The synthesized outputs are used only to support intuition and to validate internal consistency of the modeling assumptions; the quantitative evaluation in this paper is based on the controlled empirical dataset described next, with empirical images subsequently standardized in preprocessing (grayscale conversion, cropping/alignment, intensity inversion, and row-wise normalization) prior to scanline-wise analysis. Appendix D extends this illustrative construction to multi-component trajectory superpositions for additional intuition without changing the validated one-dimensional measurand.

### 2.2. Empirical Dataset Construction

The synthesis of a robust empirical dataset represents a pivotal component in establishing a quantitative foundation for motion-blur quantification under controlled conditions. As a first phase, this study deliberately employs simplified planar motion to establish fundamental principles before advancing toward more complex relative motion. This subsection outlines the systematic dataset construction, emphasizing precision in experimental variable control and rigorous ground-truth annotation that enables isolation of fundamental blur–velocity relationships.

**Figure 3:**
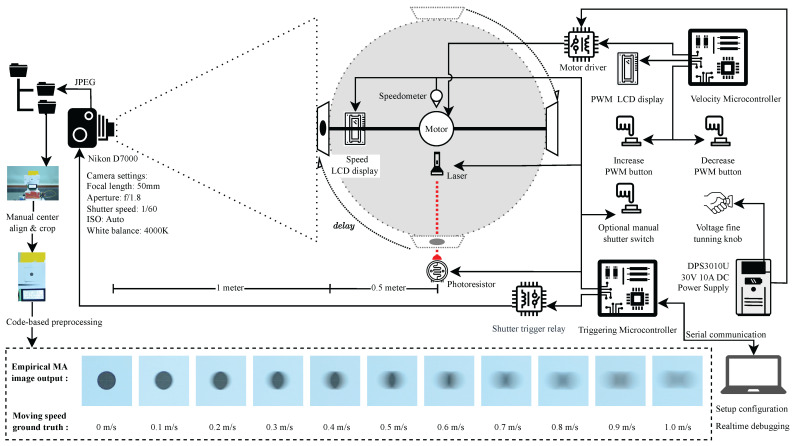
Controlled empirical image dataset construction overview (top-view).

Figure 3 illustrates the automated system constructed to provide reproducibility and high-precision control. The first part governs rotational speed, while the second orchestrates accurate shutter triggering. While purely computer-generated images can be convenient to produce, we intentionally use a physical capture setup so that the dataset reflects real sensor exposure and optics, yet still provides trustworthy ground-truth velocity labels under controlled conditions.

#### 2.2.1. Objective and Ground-Truth Determination

The principal objective of this dataset construction is to establish a direct, quantifiable relationship between the object’s true linear speed *v* and the corresponding level of motion blur observed in captured images. The velocity *v*, herein considered the independent variable, serves as the ground-truth label for each recorded image. The observable blur, representing deformation or smearing in the recorded imagery, constitutes the dependent variable. Given physical intuition, it is hypothesized that the quantity of blur should increase monotonically with *v*, enabling the inference of velocity from blur characteristics under the validated motion model.

#### 2.2.2. Velocity Control Subsystem

The rotational motion was induced via a geared brush DC motor affixed to a 0.5 m radius metallic rod. A printed marker (a black dot on white paper) was attached to one extremity of the rod, while a counterweight ensured dynamic balance. The angular motion was governed by the Velocity Microcontroller, leveraging fine-grained voltage control ( mV step) and pulse-width modulation (PWM) with an 8-bit resolution. The system supports bidirectional motion (clockwise and counterclockwise), with tunable speed increments from 0.0m/s to 1.0m/s, at intervals of 0.1m/s. The derivation of tangential velocity from angular parameters is provided in Appendix F.

#### 2.2.3. Shutter Triggering Subsystem

The image acquisition process was orchestrated by the Triggering Microcontroller, which initiated camera exposure upon detecting the motion marker’s interruption of a laser beam aimed orthogonally at a photoresistor. A precise delay interval was empirically calibrated for each velocity level to ensure image capture occurred when the moving marker was centrally located within the camera’s field of view. The display of the current velocity was embedded in each image to serve as an embedded annotation of ground truth.

Images were recorded for each predefined speed $v_{i}\in \left\{0.0,0.1,0.2,\ldots ,1.0\right\}\;m/s$, with multiple captures per speed to ensure statistical robustness, and stored in JPEG format sorted by velocity level. After acquisition, all images were manually aligned for spatial consistency: each was cropped and repositioned so that the black dot was centered horizontally and vertically aligned with a constant offset from the top edge, retaining only the central region containing the object and the numerical speed display. This preprocessing ensured that subsequent MA analysis was not confounded by translational misalignments.

### 2.3. Preprocessing

In general, even though we refer to an image as grayscale, it is often stored in a compressed JPEG format with three-channel RGB data. Therefore, it is necessary to manually check and convert such images into true grayscale before proceeding with subsequent operations. A color image (RGB) is converted to a grayscale image by computing a weighted sum of the Red, Green, and Blue channels for each pixel. The calculation proceeds as follows:

(13) $$\begin{array}{cc} I_{\mathrm{gray}}\left(x,y\right)= & 0.2989\;R(x,y)+0.5870\;G(x,y)+0.1140\;B(x,y) \end{array}$$

where $I_{\mathrm{gray}}\left(x,y\right)$ is the grayscale intensity at pixel (x,y), and R(x,y), G(x,y), and B(x,y) are the red, green, and blue channel values at pixel (x,y). These weights reflect human visual sensitivity to different colors, making the grayscale image perceptually accurate.

The dataset is organized into folders corresponding to velocity levels (i.e., speed-specific directories). For each folder *f* and base path *p*, the full path is: $D_{f}=p\;∥\;f$, where ‖ denotes path concatenation. A subset of images is selected for processing, indexed by S⊂{1,2,…,N}, where *N* is the total number of images. For each k∈S, process image $I_{k}$.

For consistent region-of-interest analysis, each image is cropped to a rectangle centered at $\left(c_{x},c_{y}\right)$ with width *w* and height *h*. Given an image of size H×W, we define the crop center as $\left(c_{x},c_{y}\right)$ and specify the crop dimensions as w×h. The resulting cropped image $I_{\mathrm{crop}}$ is obtained by extracting the region centered at $\left(c_{x},c_{y}\right)$ with the specified width and height:

(14) $$I_{\mathrm{crop}}=I\left(c_{y}-\frac{h}{2}:c_{y}+\frac{h}{2},\;c_{x}-\frac{w}{2}:c_{x}+\frac{w}{2}\right)$$

This operation symmetrically crops the grayscale image around the specified center, ensuring uniformity and alignment for subsequent analysis, and is designed to focus on regions of interest. To enforce a consistent contrast polarity and to emphasize the high-contrast marker relative to the background, pixel values are inverted:

(15) $$I_{k}^{\mathrm{inv}}\left(x,y\right)=255-I_{k}^{\mathrm{crop}}\left(x,y\right)$$

where $I_{k}^{\mathrm{inv}}\left(x,y\right)$ is the inverted pixel value at position (x,y) in the cropped image $I_{k}^{\mathrm{crop}}$. This inversion step ensures that subsequent scanline-wise signal analysis operates on a consistent intensity orientation across all samples.

Following the inversion step, each processed image $I_{k}^{\mathrm{inv}}$ is subjected to row-wise normalization to enhance the robustness and reliability of MA detection. For each horizontal scanline (row) in $I_{k}^{\mathrm{inv}}$, treated as a one-dimensional spatial signal, *z*-score normalization is applied to mitigate the influence of absolute intensity variations and global illumination changes.

Specifically, for a grayscale image $I_{k}^{\mathrm{inv}}$ of size M×N, each row *i* (1≤i≤M) is first converted to double precision. The mean $\mu_{i}$ and standard deviation $\sigma_{i}$ of row *i* are calculated as follows:

(16) $$\mu_{i}=\frac{1}{N}\sum_{j=1}^{N}I_{k}^{\mathrm{inv}}\left(i,j\right)$$

(17) $$\sigma_{i}=\sqrt{\frac{1}{N}\sum_{j=1}^{N}{\left(I_{k}^{\mathrm{inv}}\left(i,j\right)-\mu_{i}\right)}^{2}}$$

where $I_{k}^{\mathrm{inv}}\left(i,j\right)$ is the pixel value at row *i* and column *j*. The mean $\mu_{i}$ represents the average intensity of the row, while $\sigma_{i}$ quantifies its variability. The normalized value for each pixel in the row is then:

(18) $$I_{\mathrm{norm}}\left(i,j\right)=\left\{\begin{array}{cc} \frac{I_{k}^{\mathrm{inv}}\left(i,j\right)-\mu_{i}}{\sigma_{i}}, & \sigma_{i}>0,\\ 0, & \sigma_{i}=0 \end{array}\right.$$

for all j=1,…,N. Rows with $\sigma_{i}=0$ are structurally uninformative and are therefore treated as degenerate scanlines in subsequent processing. This normalization ensures that informative scanlines have zero mean and unit variance, allowing subsequent detection algorithms to focus on intrinsic signal structure rather than unrelated intensity fluctuations. As a result, artifact detection becomes more accurate and interpretable, as it is based on relative changes rather than global brightness or contrast differences.

With this row-wise normalized image $I_{\mathrm{norm}}$, we now have the essential input required for our first proposed algorithm. This preparation sets the stage for the next section, where we introduce and detail our MAPE technique—an innovative approach that leverages these normalized signals for robust artifact detection.

### 2.4. Movement Artifact Position Estimation (MAPE)

**Figure 4:**
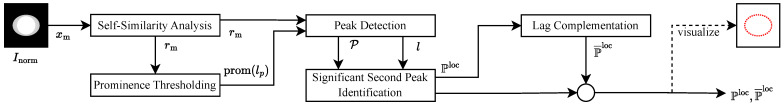
MAPE algorithm workflow.

The *MAPE* algorithm offers a structured and reproducible approach for detecting and quantifying MA in grayscale images by analyzing the spatial structure of normalized signals along horizontal scanlines. Working on the normalized image matrix $I_{\mathrm{norm}}$, MAPE follows the logical sequence summarized in Figure 4 to reliably identify artifact positions.

For computational efficiency and consistency, all major matrices and arrays required by MAPE—including the impulse response *r*, lag indices *l*, and row-wise artifact positions $p_{m}^{\mathrm{loc}}$—are preallocated to their full expected sizes.

Given a normalized image matrix $I_{\mathrm{norm}}$ of dimensions M×N, the algorithm processes each scanline by extracting, for every row *m* (where m=1,2,…,M), the signal is as follows:

(19) $$x_{m}=\left[I_{\mathrm{norm}}\left(m,1\right),I_{\mathrm{norm}}\left(m,2\right),\ldots ,I_{\mathrm{norm}}\left(m,N\right)\right]$$

This systematic enumeration of scanlines establishes a consistent foundation for subsequent impulse response analysis and artifact localization.

#### 2.4.1. Self-Similarity Analysis

For each scanline signal $x_{m}=\left[x_{m}\left[1\right],x_{m}\left[2\right],\ldots ,x_{m}\left[N\right]\right]$, compute the normalized impulse response sequence to characterize structural self-similarity across all spatial displacements.

Let the lag domain be D={−(N−1),…,N−1}. For each lag l∈D, define the overlap index set

(20) I(l)={n∈{1,…,N}:1≤n−l≤N}

so that only valid overlapping samples contribute to the response. The unnormalized impulse response at lag *l* is then

(21) $$a_{m}\left[l\right]=\sum_{n\in I(l)}x_{m}\left[n\right]\;x_{m}\left[n-l\right]$$

which is equivalent to zero-padding outside the valid index range. Appendix E presents the standard error analysis of the normalized impulse response.

Next, compute the normalization factors to scale the impulse response properly for each lag:

(22) $$\gamma_{m}\left[l\right]=\sqrt{\sum_{n\in I(l)}x_{m}{\left[n\right]}^{2}}\;\cdot \;\sqrt{\sum_{n\in I(l)}x_{m}{\left[n-l\right]}^{2}}$$

The normalized impulse response at lag *l* is then given by combining the above:

(23) $$r_{m}\left[l\right]=\left\{\begin{array}{cc} \frac{a_{m}\left[l\right]}{\gamma_{m}\left[l\right]}, & \gamma_{m}\left[l\right]>0,\\ \mathrm{NaN}, & \gamma_{m}\left[l\right]=0 \end{array}\right.\;l\in D,$$

where $r_{m}\left[0\right]=1$ for non-degenerate scanlines, providing a consistent scale for comparing similarity across lags. The resulting sequence $r_{m}=\left[r_{m}\left[-N+1\right],\;\ldots ,\;r_{m}\left[0\right],\;\ldots ,\;r_{m}\left[N-1\right]\right]$ is indexed by the lag vector $l_{m}=\left[-N+1,\;\ldots ,\;0,\;\ldots ,\;N-1\right]$. The subscript “*m*” indicates row-wise (scanline) processing. For clarity, we use $r\equiv r_{m}$ for the row-wise impulse response. This profile quantifies scanline self-similarity, with the peak at zero lag indicating perfect alignment. The sequence $r_{m}$ provides the basis for peak detection and artifact localization, supporting the prominence-based analysis in Section 2.4.3.

#### 2.4.2. Peak Detection via Prominence Criterion

For each normalized impulse response sequence r[l], significant peaks are identified using a minimum prominence threshold to ensure robustness against noise. Candidate peaks are evaluated only on the interior lag set $D^{{}^{\circ}}=\left\{-\left(N-2\right),\ldots ,N-2\right\}$, so that the immediate neighbors l−1 and l+1 are well-defined. The prominence (prom) of a candidate peak at lag $l_{p}\in D^{{}^{\circ}}$ is defined as follows:

(24) $$\mathrm{prom}\left(l_{p}\right)=r\left[l_{p}\right]-\max\left(\min_{l<l_{p}}r\left[l\right],\;\min_{l>l_{p}}r\left[l\right]\right)$$

A peak at $l_{p}$ is retained if $\mathrm{prom}(l_{p})\geq \rho$, where ρ is the minimum peak prominence, set as either a fixed value or adaptively as $\rho =\alpha \;\cdot \;\sigma_{r}$, with α a scaling parameter and $\sigma_{r}$ the standard deviation of r[l], which is computed as follows:

(25) $$\sigma_{r}=\sqrt{\frac{1}{2N-1}\sum_{l=-(N-1)}^{N-1}{\left(r\left[l\right]-\mu_{r}\right)}^{2}}$$

where

(26) $$\mu_{r}=\frac{1}{2N-1}\sum_{l=-(N-1)}^{N-1}r\left[l\right]$$

is the mean of the impulse response sequence. In the experiments reported here, a single operational prominence setting was fixed after pilot tuning so that all images could be evaluated under one reproducible configuration. The set of valid peak locations is thus:

(27) $$L_{m}=\left\{l_{p}\in D^{{}^{\circ}}\;|\;\begin{array}{c} r\left[l_{p}\right]>r\left[l_{p}-1\right],\\ r\left[l_{p}\right]>r\left[l_{p}+1\right],\\ \mathrm{prom}(l_{p})\geq \rho \end{array}\right\}$$

and the corresponding peak values are

(28) $$R_{m}=\left\{r_{m}\left[l\right]:l\in L_{m}\right\}.$$

Thus, for each scanline, the detected peaks are described by the pairs $\left\{\left(l,r_{m}\left[l\right]\right):l\in L_{m}\right\}$. This explicit association between peak values and their lag locations is essential for subsequent artifact localization and quantification. This approach ensures that only structurally significant and well-isolated peaks are selected for subsequent artifact localization.

#### 2.4.3. Identification of the Significant Second Peak

For each scanline’s similarity response profile, first identify all local maxima (peaks) that satisfy the prominence criterion established in Step 3. Exclude minor peaks attributable to noise by applying the prominence threshold. Next, sort the detected peaks in descending order according to their values to prioritize the most significant features for artifact analysis.

Let $K_{m}=\left|L_{m}\right|$ denote the total number of detected peaks for scanline *m*. Enumerate the detected lag locations as $\left[l_{m,1},l_{m,2},\ldots ,l_{m,K_{m}}\right]$ and the corresponding peak values as $\left[r_{m}\left[l_{m,1}\right],r_{m}\left[l_{m,2}\right],\ldots ,r_{m}\left[l_{m,K_{m}}\right]\right]$. Sorting the peak values in descending order yields an ordering $\pi_{m}$ such that

$$r_{m}\left[l_{m,\pi_{m}\left(1\right)}\right]\geq r_{m}\left[l_{m,\pi_{m}\left(2\right)}\right]\geq \ldots \geq r_{m}\left[l_{m,\pi_{m}\left(K_{m}\right)}\right].$$

The lag (shift) value corresponding to the second-highest peak is then defined as:

(29) $$p_{m}^{\mathrm{loc}}=l_{m,\pi_{m}\left(2\right)}$$

where $p_{m}^{\mathrm{loc}}$ denotes the lag position of the second most significant peak in the impulse response analysis for row *m*. If $K_{m}<2$, assign $p_{m}^{\mathrm{loc}}=\mathrm{NaN}$ to indicate the absence of a valid second peak.

#### 2.4.4. Symmetry-Based Lag Complementation

To retain both directional interpretations of the same wrapped displacement during pairing, we define a complementary lag for each detected second-peak location. For a nonzero lag $p_{m}^{\mathrm{loc}}\in D$, the complementary lag is the alternative representative of the same displacement modulo *N*:

(30) $${\bar{p}}_{m}^{\mathrm{loc}}=\left\{\begin{array}{cc} p_{m}^{\mathrm{loc}}+N, & \mathrm{if}\;p_{m}^{\mathrm{loc}}<0,\\ p_{m}^{\mathrm{loc}}-N, & \mathrm{if}\;p_{m}^{\mathrm{loc}}>0,\\ 0, & \mathrm{if}\;p_{m}^{\mathrm{loc}}=0 \end{array}\right.$$

This operation keeps both signed representatives of the displacement available and avoids mixing lag notation with absolute pixel coordinates.

The outputs of MAPE, namely the lag position of the second most significant peak and its complemented value, are central to the subsequent quantification process. For the full image, we collect them as

(31) $$P^{\mathrm{loc}}=\left\{p_{i}^{\mathrm{loc}}|i=1,2,\ldots ,M\right\}$$

where $p_{i}^{\mathrm{loc}}$ denotes the lag index of the second most prominent peak in the impulse response of the *i*-th scanline. Correspondingly, the set of symmetry-complemented lag positions is:

(32) $${\bar{P}}^{\mathrm{loc}}=\left\{{\bar{p}}_{i}^{\mathrm{loc}}|i=1,2,\ldots ,M\right\}$$

where ${\bar{p}}_{i}^{\mathrm{loc}}$ is computed from $p_{i}^{\mathrm{loc}}$ according to the symmetry-based transformation described above.

The sets $P^{\mathrm{loc}}$ and ${\bar{P}}^{\mathrm{loc}}$ succinctly encapsulate the spatial positions of MA and their directional complements across all scanlines, serving as critical inputs for the subsequent MAQ algorithm. By systematically examining each row’s normalized signal, the MAPE algorithm computes the impulse response (self-similarity profile) and detects significant peaks, with the second most prominent peak indicating the artifact position. A symmetry-based lag transformation is then applied to ensure a comprehensive analysis of displacement directionality. This approach enables robust localization of MA within the spatial domain and facilitates both interpretability and reproducibility. However, while the spatial positions of artifacts are now clearly identified, quantifying their displacement requires knowledge of the true point of origin—the undistorted reference position of the underlying structure. Accurate determination of this origin is essential for measuring the spatial distance between each artifact and its source, thereby enabling precise artifact quantification.

### 2.5. Reference Origin Point Estimation (ROPE)

**Figure 5:**
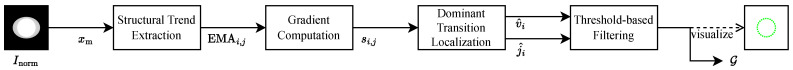
ROPE algorithm workflow.

Given a normalized image matrix $X\in R^{M\times N}$, the ROPE algorithm estimates the reference origin point for each scanline through the structured sequence of signal processing steps illustrated in Figure 5. The algorithm operates as follows:

#### 2.5.1. Structural Trend Extraction

To attenuate high-frequency noise and minor fluctuations, each scanline $x_{i}=\left[x_{i,1},x_{i,2},\ldots ,x_{i,N}\right]$ from the normalized image matrix $I_{\mathrm{norm}}$ is smoothed using an exponential moving average (EMA), recursively defined as:

(33) $$\begin{array}{cc} {\mathrm{EMA}}_{i,1} & =x_{i,1},\\ {\mathrm{EMA}}_{i,j} & =\alpha \;x_{i,j}+\left(1-\alpha \right)\;{\mathrm{EMA}}_{i,j-1},\\ \; & \;j=2,\ldots ,N \end{array}$$

where α=0.15 is the smoothing parameter. This first-order low-pass smoothing operation preserves dominant structural transitions while suppressing noise, thereby facilitating reliable trend analysis.

#### 2.5.2. Gradient Computation

The rate of change in the smoothed signal is quantified by computing the first difference (slope) of the EMA sequence:

(34) $$s_{i,j}={\mathrm{EMA}}_{i,j+1}-{\mathrm{EMA}}_{i,j},\;j=1,\ldots ,N-1$$

This highlights locations of rapid transition, which are typically associated with structural boundaries or artifact origins.

#### 2.5.3. Dominant Transition Localization

The algorithm locates the peak in the slope sequence for each row to identify the most significant transition. The direction of the initial slope determines whether the maximum or minimum is selected:

$$\left\{\begin{array}{cc} \mathrm{If}\;s_{i,1}>0: & \left({\hat{v}}_{i},\;{\hat{j}}_{i}\right)=\max_{j}\;s_{i,j}\\ \mathrm{Else}: & \left({\hat{v}}_{i},\;{\hat{j}}_{i}\right)=\min_{j}\;s_{i,j} \end{array}\right.$$

where ${\hat{v}}_{i}$ is the peak slope value and ${\hat{j}}_{i}$ is its corresponding index. This peak identifies the point of greatest structural change along the scanline and serves as the candidate reference origin for that row. *Interpretation*: The sign of the peak slope value ${\hat{v}}_{i}$ indicates the direction of the intensity transition. If ${\hat{v}}_{i}>0$, the transition is from dark to bright (increasing intensity); if ${\hat{v}}_{i}<0$, the transition is from bright to dark (decreasing intensity).

#### 2.5.4. Threshold-Based Filtering

To improve robustness, only those peaks whose magnitude exceeds a threshold are retained:

(35) $$T=\tau \;\cdot \;\max_{i}\left|{\hat{v}}_{i}\right|,\;{\hat{v}}_{i}=s_{i,{\hat{j}}_{i}}$$

(36) $${\hat{j}}_{i}=\left\{\begin{array}{cc} {\hat{j}}_{i}, & \mathrm{if}\;|{\hat{v}}_{i}|\;>T\\ \mathrm{NaN}, & \mathrm{otherwise} \end{array}\right.$$

where τ is a user-defined sensitivity parameter (default: τ=0.8), empirically determined to balance sensitivity and robustness based on iterative validation across representative datasets and then held fixed across the controlled dataset to avoid per-image retuning. The slope peak location set for the entire image is thus:

(37) $$G=\{{\hat{j}}_{i}|i=1,2,\ldots ,M\}$$

The set G encapsulates the spatial indices of the most significant transitions in the smoothed signal for each row. These indices serve as robust reference origin points, which are critical for subsequent artifact quantification and localization. By focusing on the most prominent slope transitions, the algorithm effectively isolates the structural boundaries introduced by MA, thereby enhancing the reliability and interpretability of the overall detection framework. This output is foundational for downstream analysis and is a principal contribution of the proposed method.

Through this structured sequence, the ROPE algorithm robustly identifies the dominant structural transition in each scanline, providing a stable and interpretable reference origin essential for subsequent artifact quantification. By first applying an exponential moving average to attenuate high-frequency noise, ROPE ensures that the subsequent slope calculation highlights only significant transitions in the signal. The algorithm then locates the most prominent peak in the slope sequence—corresponding to the point of greatest change—and applies a threshold to suppress weak or spurious transitions. This process yields a reliable reference origin for each scanline, forming a robust and interpretable basis for accurate MA analysis in empirical image data.

### 2.6. Movement Artifact Quantification (MAQ)

**Figure 6:**
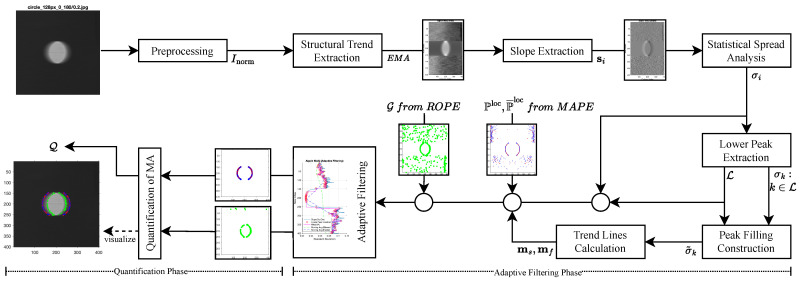
MAQ algorithm workflow.

The MAQ algorithm builds upon the outputs of the MAPE and ROPE algorithms to provide a comprehensive quantification of MA in spatial signals. Figure 6 summarizes this process. The algorithm operates on the normalized image matrix $I_{\mathrm{norm}}\in R^{M\times N}$, the set of detected artifact positions from MAPE, $P^{\mathrm{loc}}={\left\{p_{i}^{\mathrm{loc}}\right\}}_{i=1}^{M}$, their symmetry-complemented counterparts ${\bar{P}}^{\mathrm{loc}}={\left\{{\bar{p}}_{i}^{\mathrm{loc}}\right\}}_{i=1}^{M}$, and the set of estimated reference origin points from ROPE, $G={\left\{{\hat{j}}_{i}\right\}}_{i=1}^{M}$.

Although the MAPE algorithm yields the set of detected artifact positions, $P^{\mathrm{loc}}$, and the ROPE algorithm provides the set of estimated origins, G, a direct computation of the spatial displacement between these two sets is nontrivial in practice. This is due to the inherent possibility that the detected artifact positions and the estimated origins may not correspond one-to-one, and in some cases, the spatial separation $|p_{i}^{\mathrm{loc}}-{\hat{j}}_{i}|$ may be excessively large, indicating an invalid or spurious pairing. Furthermore, the MAPE algorithm produces both the set of detected artifact positions, $P^{\mathrm{loc}}$, and the set of symmetry-complemented lag positions, ${\bar{P}}^{\mathrm{loc}}$, necessitating a principled approach to select the most appropriate pairing with the origin for each scanline.

Consequently, the MAQ algorithm is structured around two critical signal processing steps: (1) a robust comparison and selection mechanism to identify the valid origin-artifact pair among the candidate sets $P^{\mathrm{loc}}$ and ${\bar{P}}^{\mathrm{loc}}$ for each row, and (2) an adaptive filtering strategy to suppress outlier or inconsistent pairings based on spatial proximity and signal characteristics. These steps ensure that the quantification of artifact displacement is both accurate and resilient to noise or structural ambiguity.

The MAQ algorithm proceeds through the following steps, using the consistent parameter notation established in the previous MAPE and ROPE algorithms:

#### 2.6.1. Adaptive Filtering Phase

We begin by quantifying the local variability of the slope signal for each spatial row. Let $S\in R^{M\times (N-1)}$ denote the matrix of slope sequences, where each row $s_{i}=\left[s_{i,1},s_{i,2},\ldots ,s_{i,N-1}\right]$ is the first-order difference of the EMA of the normalized signal. For each row *i*, we compute the standard deviation of the slope sequence, which is defined as

(38) $$\sigma_{i}=\sqrt{\frac{1}{N-2}\sum_{j=1}^{N-1}{\left(s_{i,j}-{\bar{s}}_{i}\right)}^{2}}$$

where

(39) $${\bar{s}}_{i}=\frac{1}{N-1}\sum_{j=1}^{N-1}s_{i,j}$$

This yields the vector $\sigma ={\left[\sigma_{1},\sigma_{2},\ldots ,\sigma_{M}\right]}^{⊤}$, which characterizes the spatial distribution of slope variability across the image. To identify regions of minimal variability—indicative of stable or artifact-free zones—we seek the local minima (lower peaks) in the standard deviation sequence. This is achieved by applying a peak-finding algorithm to the negated standard deviation vector, −σ, such that the set of lower peak indices is given by

(40) $$L=\left\{k:\sigma_{k}<\sigma_{k-1},\;\sigma_{k}<\sigma_{k+1}\right\}$$

and the corresponding lower peak values are $\sigma_{k}:k\in L$. If no such minima exist, the set is assigned as empty or filled with NaN for subsequent processing. Since the lower peak values may not be defined for all indices, a completed sequence $\tilde{\sigma }$ is constructed by filling each undefined entry with the nearest valid lower peak value, either to the left or right, whichever is closer. Formally, for each *k* where $\sigma_{k}$ is undefined,

(41) $${\tilde{\sigma }}_{k}=\left\{\begin{array}{cc} \sigma_{l^{*}}, & l^{*}=\arg\min_{l\in L}\left|k-l\right|\\ \mathrm{NaN}, & \mathrm{if}\;L=\emptyset \end{array}\right.$$

where $l^{*}$ is the index of the nearest lower peak to *k*. This results in a complete sequence $\tilde{\sigma }={\left[{\tilde{\sigma }}_{1},{\tilde{\sigma }}_{2},\ldots ,{\tilde{\sigma }}_{M}\right]}^{⊤}$ that captures the local variability profile across all scanlines.

Next, two moving average sequences over $\tilde{\sigma }$ are defined: a slower (less sensitive) trend line $m_{s}$ and a faster (more sensitive) trend line $m_{f}$, computed as

(42) $$m_{s}\left(i\right)=\frac{1}{w_{s}}\sum_{k=i-\lfloor w_{s}/2\rfloor }^{i+\lfloor w_{s}/2\rfloor }{\tilde{\sigma }}_{k}$$

(43) $$m_{f}\left(i\right)=\frac{1}{w_{f}}\sum_{k=i-\lfloor w_{f}/2\rfloor }^{i+\lfloor w_{f}/2\rfloor }{\tilde{\sigma }}_{k}$$

where $w_{s}$ and $w_{f}$ are the window lengths for the slow and fast moving averages, respectively, and the sums are taken over valid indices within the range 1≤k≤M. In our implementation, we set $w_{s}=100$ and $w_{f}=25$ for the fixed input height (M=400 scanlines after cropping). This choice enforces a two-scale separation: the slower window captures broader, image-level variability trends across scanlines, while the faster window responds to local fluctuations. Both values are kept fixed across all experiments to avoid per-image tuning.

The adaptive filtering criterion is then applied as follows: for each row *i*, if $m_{f,i}>m_{s,i}$, or if $\sigma_{i}>m_{s,i}$ when $m_{f,i}\leq m_{s,i}$, the corresponding slope peak location and associated second-peak candidates are set to NaN, indicating suppression due to excessive local variability or inconsistency with the global trend. This can be written as

$$\left\{\begin{array}{cc} {\hat{j}}_{i}\leftarrow \mathrm{NaN},\;p_{i}^{\mathrm{loc}}\leftarrow \mathrm{NaN},\;{\bar{p}}_{i}^{\mathrm{loc}}\leftarrow \mathrm{NaN}, & \mathrm{if}\;m_{f,i}>m_{s,i}\\ {\hat{j}}_{i}\leftarrow \mathrm{NaN},\;p_{i}^{\mathrm{loc}}\leftarrow \mathrm{NaN},\;{\bar{p}}_{i}^{\mathrm{loc}}\leftarrow \mathrm{NaN}, & \mathrm{if}\;m_{f,i}\leq m_{s,i}\;\mathrm{and}\;\sigma_{i}>m_{s,i}\\ {\hat{j}}_{i}\leftarrow {\hat{j}}_{i},\;p_{i}^{\mathrm{loc}}\leftarrow p_{i}^{\mathrm{loc}},\;{\bar{p}}_{i}^{\mathrm{loc}}\leftarrow {\bar{p}}_{i}^{\mathrm{loc}}, & \mathrm{otherwise} \end{array}\right.$$

where ${\hat{j}}_{i}$ is the filtered slope peak location and $p_{i}^{\mathrm{loc}},{\bar{p}}_{i}^{\mathrm{loc}}$ are the retained MAPE candidates for row *i*.

This procedure ensures that only reference points and artifact positions consistent with both local and global variability trends are retained, thereby enhancing MA quantification robustness. Operationally, rows rejected by this rule are treated as ambiguous origin-artifact pairings because elevated local variability or fast-trend excursions indicate unstable correspondence. The moving average of lower peaks acts as a dynamic threshold, adapting to both abrupt and gradual signal changes while suppressing outliers that deviate from expected variability. This adaptive mechanism is particularly effective in empirical datasets where noise and artifact characteristics may vary spatially across the image.

#### 2.6.2. Quantification Phase

Let $G={\left\{{\hat{j}}_{i}\right\}}_{i=1}^{M}$ denote the set of filtered slope peak locations (reference origin points) for each row *i*, and let $P^{\mathrm{loc}}={\left\{p_{i}^{\mathrm{loc}}\right\}}_{i=1}^{M}$ and ${\bar{P}}^{\mathrm{loc}}={\left\{{\bar{p}}_{i}^{\mathrm{loc}}\right\}}_{i=1}^{M}$ denote the sets of second peak locations and their symmetry-complemented counterparts, as obtained from the MAPE algorithm.

For each row *i*, the MAQ value, denoted as $q_{i}$, is defined as the spatial distance between the reference origin and the most relevant second peak location. The procedure is as follows: If either the reference origin or both second peak locations are undefined (NaN), then $q_{i}=\mathrm{NaN}$; otherwise, the MAQ is computed by selecting the second peak location (either $p_{i}^{\mathrm{loc}}$ or ${\bar{p}}_{i}^{\mathrm{loc}}$) that is closest to the reference origin:

$$q_{i}=\left\{\begin{array}{cc} {\hat{j}}_{i}-{\bar{p}}_{i}^{\mathrm{loc}}, & \mathrm{if}\;p_{i}^{\mathrm{loc}}\;\mathrm{is}\;\mathrm{NaN}\;\mathrm{and}\;{\bar{p}}_{i}^{\mathrm{loc}}\;\mathrm{is}\;\mathrm{defined}\\ {\hat{j}}_{i}-p_{i}^{\mathrm{loc}}, & \mathrm{if}\;p_{i}^{\mathrm{loc}}\;\mathrm{is}\;\mathrm{defined}\;\mathrm{and}\;{\bar{p}}_{i}^{\mathrm{loc}}\;\mathrm{is}\;\mathrm{NaN}\\ {\hat{j}}_{i}-p_{i}^{\mathrm{loc}}, & \mathrm{if}\;|{\hat{j}}_{i}-p_{i}^{\mathrm{loc}}\left|\;\leq \;\right|{\hat{j}}_{i}-{\bar{p}}_{i}^{\mathrm{loc}}|\\ {\hat{j}}_{i}-{\bar{p}}_{i}^{\mathrm{loc}}, & \mathrm{otherwise} \end{array}\right.$$

To suppress outliers, a threshold is applied: if the absolute value of $q_{i}$ exceeds a predefined threshold θ, the result is suppressed: $|q_{i}|>\theta \Rightarrow q_{i}=\mathrm{NaN}$, where θ=30, as determined by empirical observation during practical operation. This threshold acts as an operational plausibility filter: it retains pairings consistent with the controlled geometry of the dataset while suppressing outliers and spurious detections, but it is not claimed to be a globally optimal threshold for arbitrary scenes.

The final set of MAQ values for all rows is thus

(44) $$Q=\{q_{i}|i=1,2,\ldots ,M\}$$

This set Q quantifies the spatial separation between the estimated reference origin and the artifact position for each scanline, providing a robust measure of MA magnitude across the image. The use of both direct and complemented second peak locations ensures symmetry and accounts for possible ambiguity in artifact positioning, while the thresholding step suppresses implausible outliers so that only plausible artifact quantities are retained for further analysis.

### 2.7. Post-Processing

**Figure 7:**
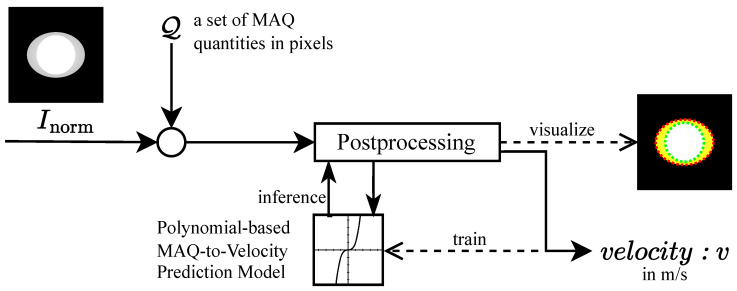
Post-processing workflow.

The post-processing workflow, illustrated in Figure 7, consists of two main phases: (1) inference, where a trained polynomial regression model maps the mean MAQ value to an estimated movement velocity, and (2) training, where the polynomial model is fitted to empirical MAQ and velocity data. This enables robust, efficient velocity estimation from artifact measurements, supporting adaptive imaging protocols and motion compensation.

#### 2.7.1. Inference Phase: Velocity Estimation from MAQ

Given a trained polynomial regression model of degree *d* with coefficients $a={\left[a_{0},a_{1},\ldots ,a_{d}\right]}^{T}$, the velocity corresponding to a new set of MAQ values is estimated as follows. Let $I_{q}=\left\{i\in \left\{1,\ldots ,M\right\}:q_{i}\;\mathrm{is}\;\mathrm{finite}\right\}$ denote the set of valid scanline-level MAQ values. The image-level summary is then

(45) $$\bar{q}=\frac{1}{|I_{q}|}\sum_{i\in I_{q}}q_{i}$$

provided that $|I_{q}|>0$; otherwise, no velocity estimate is reported for that image. The estimated velocity is then obtained by evaluating the polynomial at $\bar{q}$:

(46) $$\hat{v}=\sum_{j=0}^{d}a_{j}{\bar{q}}^{j}=a_{0}+a_{1}\bar{q}+a_{2}{\bar{q}}^{2}+\ldots +a_{d}{\bar{q}}^{d}$$

This approach provides a robust, computationally efficient mapping from the mean artifact magnitude to the estimated movement velocity. The polynomial degree *d* is typically set to 2, capturing the nonlinear relationship between MAQ and velocity while maintaining robustness and avoiding overfitting.

#### 2.7.2. Training Phase: Polynomial Model Fitting

To construct the regression model, empirical data comprising MAQ quantities and their corresponding ground-truth velocities are used. Let $Q\in R^{V\times I}$ denote the matrix of MAQ values, where *V* is the number of velocity levels and *I* is the number of images per velocity. The mean MAQ for each velocity $v_{k}$ is computed as

(47) $${\bar{q}}_{k}=\frac{1}{I}\sum_{i=1}^{I}q_{k,i},\;k=1,2,\ldots ,V$$

with the velocity vector $v={\left[v_{1},v_{2},\ldots ,v_{V}\right]}^{T}$, where $v_{k}=\left(k-1\right)\;\cdot \;\Delta v$ and Δv=0.1 m/s.

A subset of the data, $v_{\mathrm{sub}}$ and ${\bar{q}}_{\mathrm{sub}}$, is selected for model fitting. The polynomial regression problem is formulated as

(48) $$v=\sum_{j=0}^{d}a_{j}{\bar{q}}^{j}$$

with the Vandermonde matrix

(49) $$V=\left(\begin{array}{ccccc} 1 & {\bar{q}}_{\mathrm{start}} & {\bar{q}}_{\mathrm{start}}^{2} & \ldots & {\bar{q}}_{\mathrm{start}}^{d}\\ 1 & {\bar{q}}_{\mathrm{start}+1} & {\bar{q}}_{\mathrm{start}+1}^{2} & \ldots & {\bar{q}}_{\mathrm{start}+1}^{d}\\ \vdots & \vdots & \vdots & \ddots & \vdots \\ 1 & {\bar{q}}_{\mathrm{end}} & {\bar{q}}_{\mathrm{end}}^{2} & \ldots & {\bar{q}}_{\mathrm{end}}^{d} \end{array}\right)$$

The optimal coefficients are obtained by solving the least squares problem:

(50) $$a=\arg\min_{a}{∥Va-v_{\mathrm{sub}}∥}_{2}^{2}$$

with the analytical solution

(51) $$a={\left(V^{T}V\right)}^{-1}V^{T}v_{\mathrm{sub}}$$

The resulting trained model enables the inference phase described above, providing a mathematically rigorous and empirically validated mapping from MAQ quantities to velocity estimates (in m/s).

## 3. Experimental Setup

**Figure 8:**
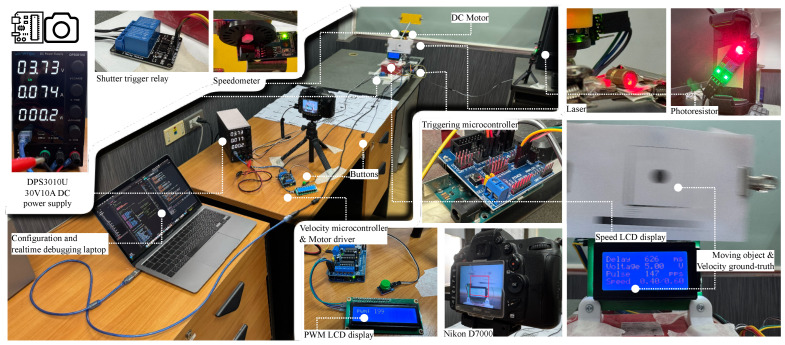
Controlled empirical image dataset construction system used in the experiment.

This section describes the controlled experimental framework used to assess the accuracy, robustness, and computational performance of the proposed motion-blur detection and quantification pipeline. We first describe the dataset construction and acquisition protocol together with the hardware and triggering apparatus (Section 3.1), including the setup shown in Figure 8. Next, we summarize the software environment and the fixed parameter settings used throughout the MAPE–ROPE–MAQ pipeline (Section 3.2). Finally, we present the evaluation protocol and metrics (Section 3.3).

### 3.1. Dataset Construction and Hardware Configuration

A custom dataset was constructed under controlled conditions to enable reproducible evaluation of motion blur across a range of object velocities. The experimental setup generated images of a high-contrast marker (a printed black dot) mounted on a rotating plate. The plate was driven to rotate with precise tangential velocities ranging from v=0.0 to 1.0 m/s, in increments of 0.1 m/s. At each speed level, ten images were captured, yielding a total of 110 empirically labeled samples. The velocity at each capture moment was displayed in an LCD integrated into the scene, serving as the embedded ground truth for downstream processing. The raw images were captured in JPEG format and stored in speed-specific directories. Figure 11e shows this directory organization. Subsequent preprocessing included manual alignment to center the marker horizontally and vertically (with a fixed offset of 720 pixels from the top edge). After the manual alignment, the images were then cropped by the code to 400 × 400 pixels before being converted to grayscale. Finally, images were inverted to enhance contrast and feature prominence in later detection stages.

The experimental equipment comprises two coordinated subsystems: (1) a velocity control unit that drives the rotating marker at prescribed tangential speeds, and (2) a triggering unit that synchronizes camera exposure to a repeatable marker position using a beam-break sensor and a calibrated delay. Key camera settings and detailed hardware architecture are reported in Appendices Appendix H and Appendix I.

### 3.2. Software and Pipeline Configuration

Data preprocessing and algorithmic implementation were performed using MATLAB R2023a and Python 3.10 with libraries such as NumPy==2.2.6, OpenCV==4.13.0.92, SciPy==1.15.3, and Matplotlib==3.10.8 for some specific plottings. The development environment included Visual Studio Code==1.114 with support for MATLAB extension and Docker-based workflow management. Microcontroller programming was carried out using the Arduino IDE, while GitHub==3.5.7 Desktop facilitated version control. Anaconda Navigator was employed for Python environment management. The experiments were executed on both an NVIDIA CUDA-enabled Windows x86_64 system and an Apple M1-based platform running under the Rosetta emulation layer.

Image preprocessing employed fixed parameters: grayscale conversion, inversion, and cropping with a width and height of 400 pixels and a vertical offset of 720 pixels. These steps ensured spatial and photometric consistency across all samples. The pipeline consists of three computational stages—MAPE, ROPE, and MAQ—with fixed parameters used for all experiments. These parameters were selected during pilot experimentation and then held constant over the full dataset to avoid per-image retuning. Table 1 summarizes the operational values and their roles.

### 3.3. Evaluation Protocol

The framework is evaluated via a multi-stage protocol covering signal clarity, detection accuracy, quantification reliability, and regression consistency. In preprocessing, image integrity is measured using contrast-to-noise ratio (CNR), as described in Appendix G, and quality loss percentage to confirm minimal structural and contrast distortion.

MAPE performance is measured via detection rate and false positive rate across scanlines, with peak prominence distribution analyzed to evaluate detection robustness.

ROPE is validated based on reference point detection rate and stability across varying object velocities, with sensitivity to slope threshold changes indicating reliability under motion-induced blur.

MAQ is assessed by computing the average displacement between MAPE and ROPE outputs, post-filtering. The validity of this measure is supported by the correlation between MAQ and ground-truth speed across all velocity levels. This relationship forms the basis for regression modeling.

Polynomial regressions are trained to map MAQ to velocity (*v*), using degree-2 and degree-3 fits under full-range (0–1.0 m/s) and constrained-range (0–0.7 m/s) configurations. The fitted curves are constructed from velocity-level mean MAQ values, and the reported $R^{2}$, RMSE, and MAE values should therefore be interpreted as descriptive fit statistics on the studied controlled dataset. Additional cross-validation summaries on image-level MAQ-derived velocity estimates are reported separately in Section 4.9 and Figure 17 to assess regression stability on the available data. These checks are distinct from the preprocessing CNR and quality-loss statistics in Section 4.3 and Figure 12, which quantify signal preservation rather than model generalization. Model accuracy is further evaluated using residual trends, extrapolation behavior, and fit quality.

To characterize the controlled validation boundary under stronger perturbation, an additional robustness experiment injects fixed zero-mean Gaussian noise into the cropped-image inputs across a wide noise ladder. That analysis is summarized in Section 4.11 using measured noise strength, MSE, PSNR, SNR, stage-wise valid ratios, image-level MAQ drift, and failure behavior, with the full mathematical definitions collected in Appendix J. Finally, system-level performance is reviewed through computational latency and bias-variance analysis, ensuring the proposed pipeline is accurate, stable, and interpretable across experimental conditions.

## 4. Experimental Results and Analysis

**Figure 9:**
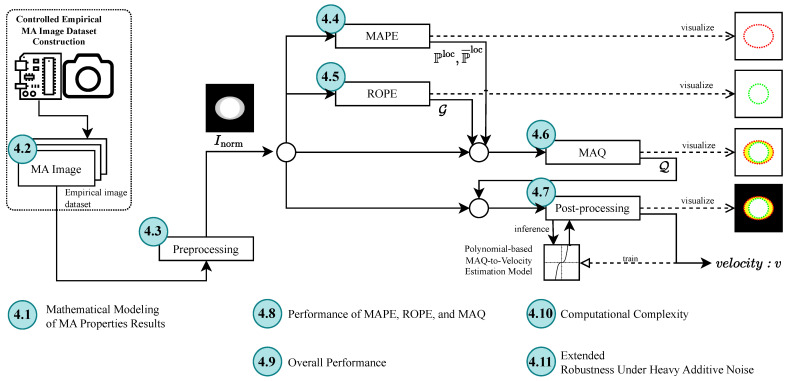
Results and analysis overview following Figure 2 and summarizing the subsequent evaluation stages.

This section presents the experimental results and in-depth analysis for each stage of the proposed MA Detection and Quantification pipeline. Figure 9 summarizes the sequence of topics analyzed in this section. The analysis spans from the validation of the mathematical model through synthetic and empirical images to the final regression-based velocity estimation. The findings validate the feasibility and performance of the proposed system under controlled empirical conditions.

### 4.1. Results of Mathematical Modeling of MA Properties

**Figure 10:**
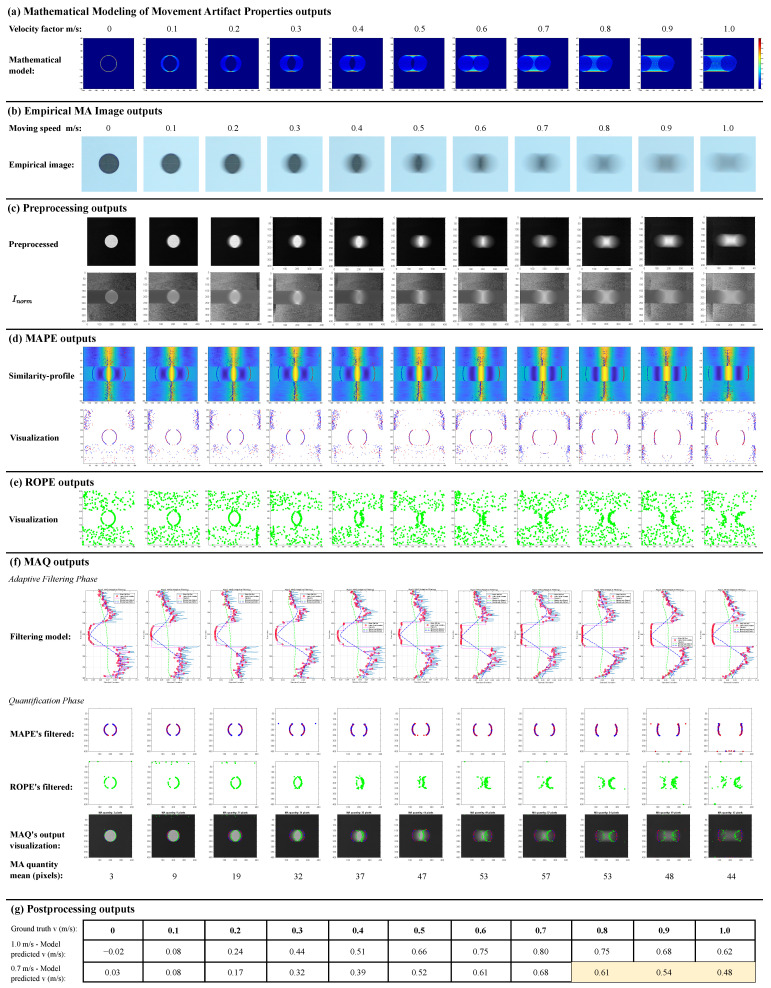
Representative visual outputs for (**a**) mathematical modeling of motion-blur properties, (**b**) empirical dataset construction, (**c**) preprocessing, (**d**) MAPE, (**e**) ROPE, (**f**) MAQ, and (**g**) post-processing.

To validate the theoretical basis of MA, we synthesized images by simulating object motion during sensor exposure. Figure 10a shows a monotonic increase in MA-induced intensity deformation over v∈[0.0,1.0]m/s: boundaries remain sharp at v=0, while noticeable distortion emerges by v=0.2m/s, with progressive blurring at higher velocities. Although these synthesized images are not used in the computational pipeline, they support the physical interpretation and correctness of the MA generation model.

### 4.2. Empirical Dataset Results

**Figure 11:**
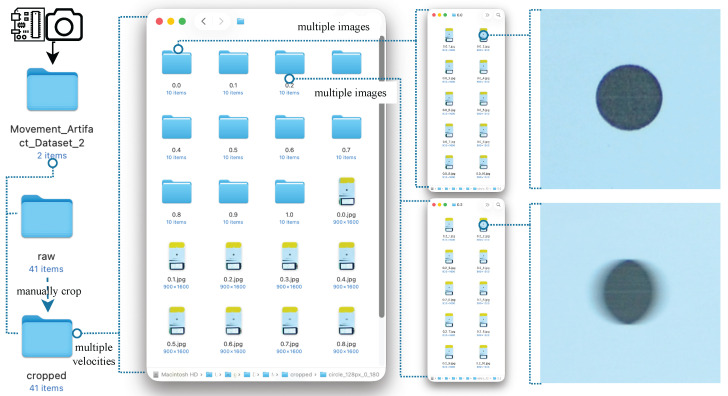
Dataset file structure and sample images.

Empirical image acquisition followed the controlled protocol described earlier. The dataset comprises 110 images stored in folders corresponding to velocity levels from v=0.0 to 1.0m/s, in steps of 0.1m/s. Each folder contains 10 images, forming a consistent and well-labeled dataset structure. Figure 11 depicts the folder organization, and Table 2 summarizes the dataset.

At zero velocity, images exhibit high edge clarity with low-frequency random noise (non-MA), as shown in the top-right image. In contrast, images captured at v=0.2m/s exhibit visible MA in the form of spatial duplication and horizontal smearing due to lateral object motion, as seen in the bottom-right image. Full image series for all speed levels are presented in Figure 10b, affirming the reliability of the dataset in reflecting MA progression.

### 4.3. Preprocessing Results

The preprocessing phase transforms raw images into standardized input signals while preserving essential structural features. In particular, it enforces a consistent contrast polarity so that prominent structures map to higher intensity, while more homogeneous regions map to lower intensity. Figure 10c shows the resulting standardized signal representation.

**Figure 12:**
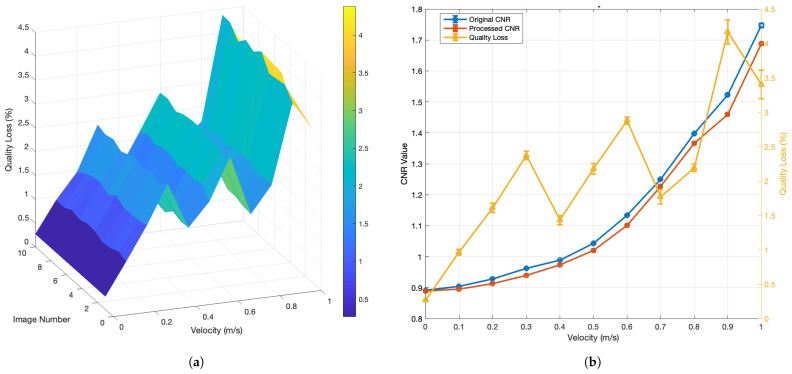
Preprocessing evaluation: (**a**) quality-loss percentage and (**b**) statistical summary of CNR and quality loss.

To quantify information preservation, we computed the contrast-to-noise ratio (CNR) before and after preprocessing at each velocity level. Figure 12a reports the percentage quality loss, and Figure 12b summarizes the corresponding CNR and quality-loss statistics. Overall, the CNR of the preprocessed images is only slightly reduced relative to the raw inputs, and the average quality degradation remains between 0.25% and 4.25%, indicating that the preprocessing introduces negligible distortion.

### 4.4. MAPE Results

The MAPE algorithm successfully identifies probable MA positions along scanlines. The output is visualized in Figure 10d using red and blue dots to represent MA detected in leftward and rightward directions, respectively. These directions correspond to negative and positive shifts in self-similarity, reflecting actual object movement directionality.

**Figure 13:**
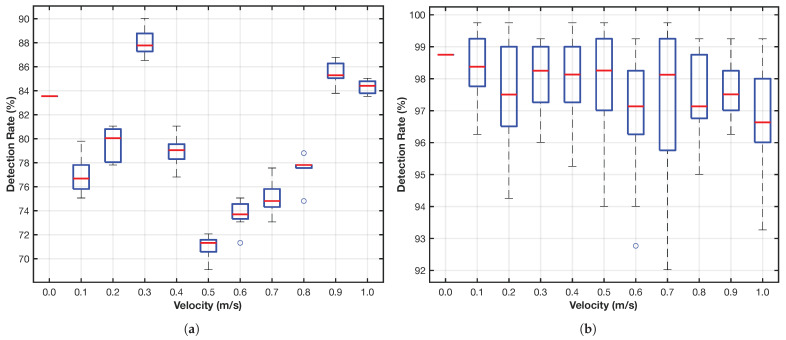
Detection-rate evaluation for (**a**) MAPE and (**b**) ROPE.

Detection performance was measured via the detection rate, defined as the ratio of correctly identified MA points to total evaluated scanlines. Figure 13a shows detection rates ranging from 70% to 90% across all velocities, indicating high robustness and stability of MAPE. Importantly, the detection rate appears uncorrelated with velocity, which supports the system’s consistency.

Some false positives were observed in homogeneous image regions lacking objects. These spurious detections require filtering, motivating the need for the ROPE and MAQ stages.

### 4.5. ROPE Results

The ROPE algorithm identifies the original location of the object prior to motion. The results are shown in Figure 10e, where green dots represent the estimated reference points distributed across the image domain.

Figure 13b shows that the ROPE detection rate, computed as the ratio of successfully localized origins to total length of scanlines, remains exceptionally high—peaking at 99% at low speeds and decreasing slightly to 97% at v=1.0m/s. This trend indicates that high-speed motion introduces slight challenges in reference localization, though performance degradation is minimal within the tested range.

### 4.6. MAQ Results

**Figure 14:**
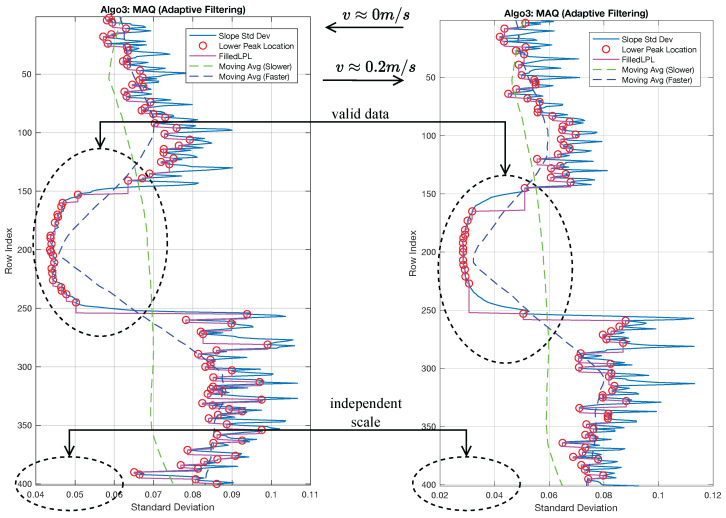
MAQ adaptive filtering models at velocity levels 0.0 and 0.2 m/s (left and right, respectively).

The MAQ algorithm computes the spatial displacement between artifact positions detected by MAPE and reference origins estimated by ROPE. To address noise and variability in both outputs, an adaptive filtering mechanism is applied prior to quantification. The resulting filtering models are illustrated in Figure 14, with criteria and thresholds dynamically adjusted for each velocity level and image. Comprehensive visualizations for all velocities are presented in the first row of Figure 10f; the second and third rows display the filtered outputs from MAPE and ROPE, respectively, while the fourth row shows the superimposed quantification result on the preprocessed image.

The MAQ algorithm produces a single scalar per image, defined as the mean artifact displacement over the retained high-confidence scanlines (in pixels). Aggregating these values across the dataset reveals a clear dependence of MAQ on object velocity: MAQ increases approximately linearly over v∈[0,0.7]m/s, spanning roughly 0–70 pixels, whereas performance degrades for v>0.7m/s, where stronger blur reduces the structural cues available for reliable quantification.

To map between MAQ and velocity, we fit two polynomial regression models: a forward model (velocity-to-MAQ) that predicts expected MAQ given *v*, and an inverse model (MAQ-to-velocity) that estimates movement speed directly from measured MAQ values.

### 4.7. Post-Processing Results

**Figure 15:**
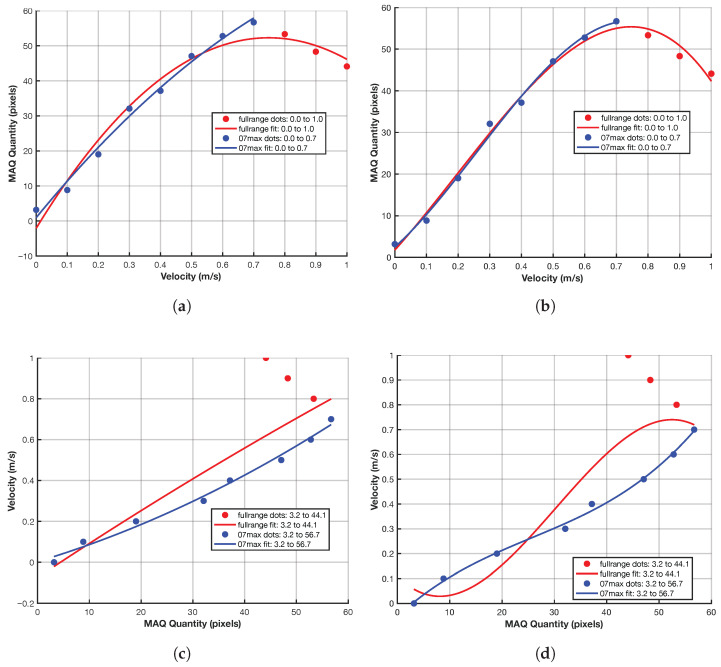
Polynomial regression analysis: (**a**) MAQ quantity versus velocity (quadratic fit), (**b**) MAQ quantity versus velocity (cubic fit), (**c**) velocity versus MAQ quantity (quadratic fit), and (**d**) velocity versus MAQ quantity (cubic fit).

Figure 15 presents polynomial fits of degree 2 and 3 for both models. The models trained over the full velocity range (v∈[0.0,1.0]) are labeled *fullrange* (red), while models trained on v∈[0.0,0.7] are labeled *07max* (blue). Figure 10g summarizes the corresponding post-processing outputs. Velocity estimates are most accurate within the 0–0.7 m/s interval. Beyond this, model accuracy diminishes, and higher-degree polynomials introduce overfitting without meaningful performance gain. A detailed analysis of the polynomial MAQ-to-velocity regression model, which operationalizes the mapping from artifact quantification to estimated movement speed, is presented in the following Section 4.9. This key result demonstrates how MA can be robustly detected and quantified to enable velocity estimation and forms the foundation for the subsequent discussion and system evaluation.

### 4.8. Performance of MAPE, ROPE, and MAQ

The comprehensive evaluation framework establishes a multi-dimensional assessment of the sequential algorithm performance through statistical normalization and weighted scoring. For each algorithm k∈{1,2,3}, which are MAPE, ROPE, and MAQ, respectively, the bias normalization is computed as

(52) $${\mathrm{norm}\text{\_}\mathrm{bias}}_{k}=\frac{|{\bar{b}}_{k}|-\min_{j}\left(\right|{\bar{b}}_{j}\left|\right)}{\max_{j}\left(\right|{\bar{b}}_{j}\left|\right)-\min_{j}\left(\right|{\bar{b}}_{j}\left|\right)}$$

where ${\bar{b}}_{k}$ represents the mean bias across velocities for algorithm *k*. Similarly, variance normalization follows

(53) $${\mathrm{norm}\text{\_}\mathrm{var}}_{k}=\frac{{\bar{\sigma }}_{k}^{2}-\min_{j}\left({\bar{\sigma }}_{j}^{2}\right)}{\max_{j}\left({\bar{\sigma }}_{j}^{2}\right)-\min_{j}\left({\bar{\sigma }}_{j}^{2}\right)}$$

The combined performance score integrates these metrics through a weighted linear combination

(54) $$S_{k}=0.4\;\cdot \;{\mathrm{norm}\text{\_}\mathrm{bias}}_{k}+0.4\;\cdot \;{\mathrm{norm}\text{\_}\mathrm{var}}_{k}+0.2\;\cdot \;\left(1-{\bar{V}}_{k}\right)$$

where ${\bar{V}}_{k}$ represents the mean validity ratio. The coefficient of variation for consistency assessment is defined as

(55) $$CV_{k}=\frac{\mathrm{std}(b_{k})}{|{\bar{b}}_{k}|}$$

where $b_{k}$ is the bias vector across all velocity-image combinations. The root mean square error combines bias and variance components as

(56) $${\mathrm{RMSE}}_{k}=\sqrt{{\bar{b}}_{k}^{2}+{\bar{\sigma }}_{k}^{2}}\;$$

where ${\bar{\sigma }}_{k}^{2}$ is the mean variance across velocities.

**Figure 16:**
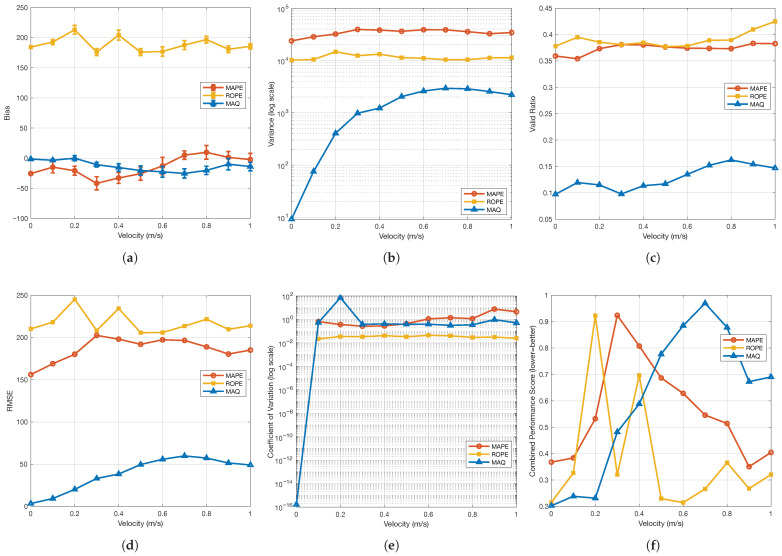
Statistical evaluation of MAPE, ROPE, and MAQ: (**a**) algorithm bias vs. velocity, (**b**) algorithm variance vs. velocity, (**c**) algorithm validity ratio vs. velocity, (**d**) root mean square error vs. velocity, (**e**) algorithm consistency vs. velocity, and (**f**) overall algorithm performance vs. velocity.

The sequential algorithm evaluation, shown in Figure 16, highlights the operational characteristics of each framework component. MAPE’s bias analysis reveals velocity-dependent performance, with optimal artifact position estimation at 0.7–0.8 m/s, where the U-shaped bias profile indicates peak precision for distinct periodic movement patterns. Its moderate validity ratio (0.69–0.90) reflects selective detection, as stringent peak criteria improve accuracy at specific velocities but reduce data yield. ROPE maintains consistently high positive bias (167.28–225.44 pixels) and exceptional validity ratios (>0.93) across all velocities, demonstrating robust, stable reference origin estimation. ROPE’s lower variance (2–3 times less than MAPE) confirms its precision in reference point detection. MAQ achieves superior bias performance (−1.44 to −37.32 pixels) via adaptive filtering, which suppresses unreliable position pairs. Its low validity ratio (0.07–0.19) indicates stringent quality control, filtering out 80–90% of scanlines and retaining only the most reliable artifact–reference pairs. Accordingly, the image-level MAQ value should be interpreted as a filtered high-confidence summary rather than an exhaustive average over all rows. MAQ’s variance increases with velocity, reflecting greater complexity at higher speeds, but overall bias remains lower than MAPE and ROPE. Despite varying individual metrics, the integrated pipeline transforms these outputs into robust velocity estimates within the validated operating regime.

The following comprehensive evaluation of the proposed MA detection and quantification framework culminates in the polynomial regression analysis that establishes the fundamental relationship between MAQ quantities and actual movement velocities.

### 4.9. Overall MA Detection and Quantification Performance

This final assessment evaluates the end-to-end algorithmic pipeline with respect to the primary research question: *how can we effectively detect and quantify MA in imaging systems?*

Figure 15 reveals two distinct operational regimes for MA quantification. The full-range polynomial model, spanning velocities from 0.0 to 1.0 m/s with MAQ values from 3.2 to 44.1 pixels, achieves an $R^{2}=0.7381$ with RMSE of 0.1618 m/s and MAE of 0.1268 m/s. The polynomial coefficients [−0.000025,0.016817,−0.073966] define the quadratic relationship:

(57) $$v=-0.000025\;\cdot \;{\mathrm{MAQ}}^{2}+0.016817\;\cdot \;\mathrm{MAQ}-0.073966$$

For applications requiring higher precision within the lower velocity range (0.0–0.7 m/s), the restricted polynomial model demonstrates substantially stronger fit quality with $R^{2}=0.9900$, RMSE of 0.0229 m/s, and MAE of 0.0221 m/s. The corresponding polynomial relationship

(58) $$v=0.000075\;\cdot \;{\mathrm{MAQ}}^{2}+0.007601\;\cdot \;\mathrm{MAQ}+0.002876$$

covers MAQ values from 3.2 to 56.7 pixels, providing superior accuracy for moderate movement scenarios.

The opposite signs of the quadratic coefficients in the full-range and restricted-range fits indicate that a single low-order polynomial does not capture the entire 0.0–1.0 m/s regime as consistently as it captures the lower-speed regime. In practice, this means that the 0.0–0.7 m/s model should be regarded as the primary calibrated model, while the full-range fit is a descriptive summary of the broader trend rather than the preferred operating model.

**Figure 17:**
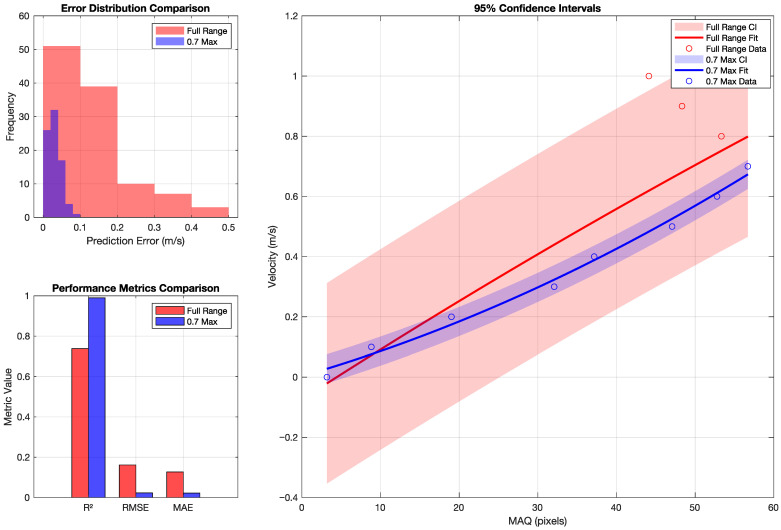
Error distribution, performance metrics, and confidence intervals for polynomial regression models.

Cross-validation summaries on the available dataset further support the relative stability of both models, with the full-range model exhibiting CV-MAE of 0.1630 m/s and maximum error of 0.4479 m/s, while the restricted model achieves CV-MAE of 0.0392 m/s with maximum error limited to 0.0794 m/s. These summaries assess the stability of the regression stage on the available image-level MAQ estimates and should be interpreted separately from the preprocessing preservation statistics reported earlier. The estimation accuracy assessment on individual MAQ measurements indicates that 95% of velocity predictions fall within ±0.3774 m/s for the full-range model and ±0.0628 m/s for the restricted model on this dataset, providing empirical uncertainty ranges for the calibrated models. The detailed error distributions, performance metrics, and confidence intervals for both models are visualized in Figure 17.

The interpolation and extrapolation capability analysis indicates that both models provide interpolation behavior consistent with their training data, with the restricted model offering the more accurate calibrated mapping for MAQ values up to 56.7 pixels. Beyond the training ranges, extrapolation can remain physically plausible but is outside the validated regime and should be interpreted cautiously until supported by additional evaluation data.

This polynomial regression framework represents the culmination of the sequential MAPE-ROPE-MAQ algorithmic pipeline, transforming raw image data through artifact position detection, reference point estimation, and adaptive quantification into calibrated velocity estimates. Within the lower-speed validated regime, the achieved sub-0.1 m/s error for moderate movement scenarios shows that the proposed framework can support quantitative MA analysis under the controlled experimental conditions studied here. The established MAQ-to-velocity relationship is therefore best interpreted as a calibrated measurement rule for this operating regime rather than as a universal model for arbitrary imaging environments.

The experimental results provide a quantitative basis for motion-aware imaging systems under the validated operating conditions. The integration of self-similarity analysis, reference origin estimation, and adaptive quantification yields a pipeline that detects, localizes, and quantifies motion blur, supporting velocity estimation with sub-0.1 m/s error for moderate speeds in the 0.0–0.7 m/s regime.

Empirical validation on a controlled dataset confirms the reliability of the proposed approach, with the polynomial relationship between artifact magnitude and movement velocity enabling practical offline analysis and calibration. These findings provide the foundation for the subsequent computational complexity analysis of pipeline scalability and efficiency.

The next subsection provides a detailed analysis of the computational complexity for each stage of the proposed MA Detection and Quantification pipeline, offering insights into scalability and performance characteristics.

### 4.10. Computational Complexity

This subsection analyzes the computational complexity of the proposed MA Detection and Quantification pipeline. Using Big-O notation, we characterize the asymptotic growth of time and space requirements with respect to input size, providing a general view of scalability. In practice, the measured processing time depends on implementation details and the performance characteristics of the underlying hardware, and may therefore vary across systems.

MAPE: For an image of size M×N, the self-similarity analysis computes the normalized impulse response for each scanline, requiring $O(N^{2})$ operations per row due to convolution-like computation across all possible lags. With *M* scanlines, the total complexity is $O(M\;\cdot \;N^{2})$. Peak detection adds O(N) operations per row, dominated by self-similarity computation.

ROPE: The most computationally efficient stage, performing exponential moving average smoothing, gradient computation, and peak detection in O(N) time per scanline. Total complexity: O(M·N).

MAQ: Combines MAPE and ROPE outputs with adaptive filtering. Standard deviation computation and moving averages require O(M) operations across scanlines, with the quantification phase performing distance calculations in O(M) time. Overall complexity: O(M·N).

Post-processing: Inference phase computes mean MAQ values in $O(N_{q})$ time and evaluates polynomial in O(d) time, where *d* is typically 2–3. The training phase performs regression fitting in O(V) time for *V* velocity levels.

Performance Implications: The end-to-end pipeline complexity is dominated by the MAPE stage, yielding $O(M\;\cdot \;N^{2})$ overall complexity. While this introduces quadratic scaling with image width, the row-independent nature enables efficient parallelization. The memory footprint remains linear in image size across all stages, supporting efficient implementations with appropriate parallel hardware acceleration, as summarized in Table 3.

Practical Considerations: The quadratic complexity of MAPE makes parallel acceleration advantageous for larger images, while ROPE and MAQ remain efficient on standard CPUs. Table 4 summarizes the pipeline’s scalability, parallelization, and optimization strategies. These results provide a basis for practical implementation and future optimization.

### 4.11. Extended Robustness Under Heavy Additive Noise

To make the empirical validation boundary more explicit, we performed an extended perturbation study in which zero-mean additive Gaussian noise was applied to the cropped-image inputs across normalized standard deviations

$$\begin{array}{cc} \sigma_{n}\in & \{0.000,0.005,0.010,0.020,0.030,0.050,0.080,\\ & 0.120,0.160,0.200,0.240,0.280,0.320,0.360,0.400\} \end{array}$$

with 10 Monte Carlo repeats per level over 11 representative images. This analysis is not intended to redefine the primary 0.0–0.7 m/s calibration regime. Instead, it stress-tests how the same scanline-domain MAPE–ROPE–MAQ formulation behaves once the marker is progressively obscured. Formal metric definitions and supplementary supporting figures are provided in Appendix J.

**Figure 18:**
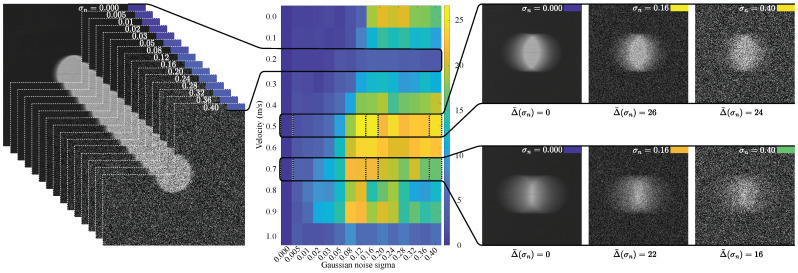
Mean absolute MAQ drift from the clean baseline across additive-noise levels.

Figure 18 shows how far the noisy image-level MAQ estimate moves away from its clean baseline as the perturbation level increases. Across the tested subset, the clean images yielded a mean MAQ of 32.36 pixels. At an intermediate perturbation level ($\sigma_{n}=0.120$), the mean absolute MAQ drift reached 14.97 pixels, while the mean MAQ valid ratio fell to 0.036. At the highest tested perturbation ($\sigma_{n}=0.400$), where the marker became barely observable, the mean MAPE, ROPE, and MAQ valid ratios were 1.000, 0.695, and 0.041, respectively, with a mean PSNR of 10.67 dB and zero recorded numerical failures.

**Figure 19:**
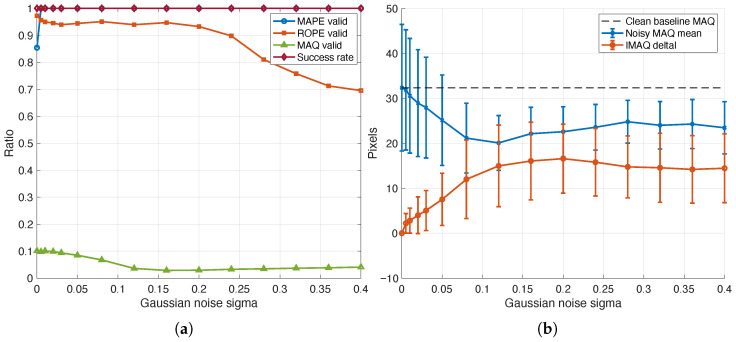
Condensed extended-robustness summary: (**a**) retention and success metrics and (**b**) image-level MAQ response under increasing additive noise.

In Figure 19a, the three stages do not degrade uniformly. Candidate MAPE detections remain relatively permissive even in the heavier-noise regime, whereas ROPE retention and especially MAQ retention become progressively more selective as structural transitions weaken and stable origin–artifact pairings become sparse. In Figure 19b, the image-level MAQ response becomes increasingly dispersed and weakly supported as noise grows. Taken together with the drift trend in Figure 18, these results show that severe additive interference can preserve candidate self-similarity responses while substantially destabilizing downstream pairing and quantification. The robustness study, therefore, sharpens the practical boundary of the present controlled single-marker regime without being interpreted as proof of robustness to arbitrary textured backgrounds, lower-contrast targets, or uncontrolled natural scenes.

## 5. Discussion

### 5.1. Performance Analysis

The experimental results validate the effectiveness of the integrated MAPE–ROPE–MAQ pipeline in quantifying motion blur with sub-0.1 m/s accuracy for velocities up to 0.7 m/s under the validated operating condition. The achieved RMSE of 0.0229 m/s for the restricted velocity range supports the use of the proposed framework as a calibrated estimator for velocity from blur quantification on the controlled dataset. These regime-specific calibration results are documented most directly in Section 4.9, together with Figure 15 and Figure 17.

The extended additive-noise analysis in Section 4.11 complements this calibration picture by showing that stronger perturbation primarily degrades ROPE retention and MAQ pairing before the pipeline collapses numerically. This distinction is useful because it separates two different limits of the current study: the velocity-calibration limit at higher blur magnitudes and the perturbation-tolerance limit under increasingly corrupted cropped inputs.

The self-similarity analysis approach employed in MAPE offers distinct advantages over purely frequency-domain signatures. While Fourier-based intuition can be helpful for understanding blur-induced changes in spatial frequency content [45], frequency-only methods can struggle with irregular or transient motion patterns. A direct benchmark against a standard frequency-domain estimator was not included in the present study because many such methods optimize different outputs—for example, global blur scores, spectral nulls, or restoration quality—rather than the calibrated scanline displacement used here. A fair head-to-head comparison would therefore require matched estimands, preprocessing assumptions, and calibration targets. Our impulse response analysis captures motion signatures through peak prominence detection, achieving detection rates of 70–90% across all tested velocities without prior knowledge of motion characteristics.

The ROPE algorithm’s reference point estimation demonstrates exceptional stability (greater than 97% detection rate). The exponential moving average approach with adaptive thresholding provides robust performance under varying noise conditions, addressing a key limitation of gradient-based techniques that suffer from sensitivity to local intensity variations.

### 5.2. Methodological Contributions

The integration of three complementary algorithms represents a novel approach to motion-blur analysis. Previous work has typically focused on either detection or compensation, but rarely addresses the quantification aspect essential for measure-first pipelines. Our framework bridges this gap by providing interpretable metrics that directly relate blur magnitude to movement velocity under controlled assumptions.

The symmetry-based lag complementation in MAPE ensures comprehensive directional analysis, addressing a limitation of conventional correlation-based methods that may miss artifacts in specific orientations. The adaptive filtering mechanism in MAQ, utilizing moving averages of variability profiles, represents an innovative approach to outlier suppression that maintains sensitivity to genuine artifacts while rejecting spurious detections.

The polynomial regression framework establishing the MAQ-to-velocity relationship provides a compact calibration rule for efficient motion estimation in the validated regime. The demonstrated $R^{2}$ = 0.99 for the restricted range indicates strong predictive alignment on the controlled dataset and motivates subsequent evaluation in broader acquisition conditions before any wider deployment claims are made.

### 5.3. Practical Implications

The proposed framework establishes foundational principles for motion-blur quantification that may contribute to imaging systems requiring objective quality assessment and motion-aware processing. As a proof-of-concept for one-dimensional horizontal motion blur, this work provides theoretical groundwork that could support future adaptive acquisition protocols and quality assurance workflows. This staged progression has already begun in a subsequent controlled direction–estimation extension built on MAPE–ROPE–MAQ outputs [67]. However, broader application remains the subject of future research phases rather than immediate deployment, as additional validation would be required to bridge the gap between controlled laboratory conditions and complex environments where multi-dimensional, non-rigid motion patterns predominate.

### 5.4. Limitations and Considerations

This study deliberately employs a controlled dataset with simplified high-contrast targets and a uniform background to establish fundamental principles for motion-blur quantification under controlled conditions. As a first phase, this approach enables rigorous validation of measurement foundations before advancing toward complex, uncontrolled scenarios. The framework focuses specifically on uniform, planar motion blur to isolate fundamental blur–velocity relationships, representing an essential methodological step toward handling multi-dimensional movements in future research phases. The extended additive-noise study in Section 4.11 partially broadens this picture by quantifying behavior under progressively corrupted cropped inputs, but it still operates within the same controlled single-marker setting. Accordingly, the present validation does not yet establish robustness to textured backgrounds, lower-contrast markers, or uncontrolled natural scenes; those broader robustness conditions require separate experiments beyond the current dataset.

Accuracy diminishes for velocities above 0.7 m/s, indicating challenges in quantifying highly blurred artifacts, where structural features become increasingly difficult to distinguish and analyze reliably. In this higher-speed regime, the blur extent broadens the marker response and weakens the distinct local transitions required by both second-peak detection and reference–origin localization; accordingly, the 0.0–0.7 m/s model should be regarded as the primary calibrated regime, while the full-range fit is descriptive only. This transition is visible in the MAQ and overall regression results reported in Section 4.6 and Section 4.9, Figure 15 and Figure 16.

The computational complexity of impulse response calculation scales quadratically with scanline length, though the row-independent processing enables efficient parallel implementation for practical deployment scenarios.

The present study fixes all operational parameters after pilot experimentation so that the full dataset can be evaluated under a single, reproducible configuration. This choice isolates the behavior of the proposed measurement pipeline, but it does not yet quantify how sensitive the final MAQ-to-velocity calibration is to individual parameter choices such as the MAPE prominence threshold ρ, the ROPE smoothing factor α, the ROPE threshold τ, the MAQ displacement threshold θ, or the moving-average window lengths $\left(w_{s},w_{f}\right)$. The fixed-parameter design, therefore, supports reproducibility, but it should not be interpreted as a proof of global optimality.

In a subsequent sensitivity and ablation study, these parameters should be perturbed one at a time and in selected joint combinations while keeping the acquisition protocol fixed. The main evaluation targets should include scanline-level detection rates, retained-scanline ratios, image-level MAQ dispersion, and changes in the restricted-range calibration metrics (RMSE, MAE, and cross-validation error). Such an analysis would separate parameters that primarily affect robustness from those that materially alter the MAQ-to-velocity mapping, thereby clarifying which values are structurally important and which are merely convenient defaults for the current dataset.

### 5.5. Future Works and Research Directions

Building on the current framework, the immediate next research direction is blur direction estimation. By determining both the magnitude and orientation of artifacts, the system can characterize complex motion patterns beyond the current horizontal focus. A direction–estimation extension has been reported separately [67]; that follow-on study preserves the same controlled empirical philosophy while extending the present measure-first pipeline from magnitude estimation toward orientation estimation.

Following direction estimation, both quantified MA magnitude and directional information will be integrated into a novel modified-DFT-based compensator. This compensator will leverage these guided parameters to perform targeted MA correction, enabling precise image quality restoration compared to conventional compensation methods that lack motion-specific guidance.

**Figure 20:**
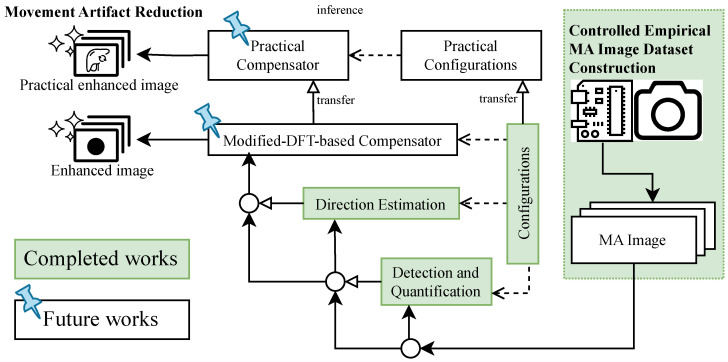
Future research roadmap for the Movement Artifact (MA) reduction module (historical system context).

The final step applies the complete pipeline—blur quantification, direction estimation, and DFT-based compensation—to real-world image datasets to assess effectiveness in realistic scenarios and evaluate robustness for motion compensation. Figure 20 summarizes the broader application roadmap (including original system context). Readers interested in the original medical-imaging pipeline context that motivated this roadmap can consult Appendix A.

## 6. Conclusions

Motion-induced artifacts (motion blur) appear as structured distortions in single-frame images when there is relative motion between the scene and the sensor. Under uniform planar motion, these distortions exhibit repeatable spatial patterns that can be detected and quantified before compensation. This work shows that blur magnitude and motion velocity can be estimated from static grayscale images using spatial-domain signal processing under controlled motion assumptions.

We introduced a complex-exponential illustrative framework to provide an analytical basis for artifact formation. The proposed MAPE–ROPE–MAQ pipeline achieved artifact detection rates of 70–90% with reference point estimation stability exceeding 97% across all tested velocities. Within the validated regime of 0.0–0.7 m/s, the polynomial regression model relating quantified displacement to motion velocity attained $R^{2}=0.99$ and RMSE =0.0229 m/s, supporting sub-0.1 m/s velocity estimation accuracy for moderate movement scenarios.

Empirical evaluation on 110 images with ground-truth velocities from 0.0 to 1.0 m/s served as the primary validation under controlled real-image acquisition conditions. An additive-noise robustness study further showed that severe perturbation can preserve candidate self-similarity responses while progressively destabilizing ROPE retention and MAQ pairing, clarifying the empirical boundary of the current controlled single-marker regime. The mathematical simulations were used only to illustrate the artifact formation mechanism and to check the internal consistency of the complex-exponential and spatial projection formulation. The self-similarity analysis, reference origin estimation, and adaptive quantification mechanisms transfer beyond synthetic examples; however, for v>0.7 m/s, stronger blur reduces structural cues and limits reliable quantification.

This framework operates under controlled imaging conditions (high-contrast targets, uniform backgrounds, and uniform planar motion) and represents an initial phase toward broader real-world applications. The $O(M\;\cdot \;N^{2})$ self-similarity computation in MAPE dominates computational complexity, but row-independent processing enables efficient parallelization. Future work extends this foundation through directional blur estimation [67], modified DFT-based compensation, and validation on real-world imaging datasets with multi-dimensional, non-rigid motion patterns characteristic of complex scenarios.

## Figures and Tables

**Table 1:** Fixed pipeline parameters used throughout the experiments.

Stage	Symbol	Value	Type	Role and Rationale
Preprocessing	w,h	400, 400	Fixed ROI	Keeps a centered, size-controlled region of interest across all samples.
Preprocessing	vertical offset	720 px	Fixed ROI	Preserves a consistent vertical placement after manual alignment.
MAPE	ρ	0.1	Empirical	Minimum prominence chosen to suppress weak secondary peaks while preserving stable detections on the controlled dataset.
ROPE	α	0.15	Empirical	Moderate EMA smoothing used to attenuate fine-scale fluctuations without erasing the dominant structural transition.
ROPE	τ	0.8	Empirical	Retains only strong slope extrema relative to the global peak magnitude in each image.
MAQ	θ	30 px	Empirical	Removes implausible origin-artifact pairings with unusually large displacement.
MAQ	$w_{s},w_{f}$	100, 25	Empirical	Implements a two-scale filter on the row-variability profile for the fixed image height M=400.

**Table 2:** Summary of the empirical image dataset.

Attribute	Value
Velocity range	0.0 to 1.0 m/s
Velocity step size	0.1 m/s
Velocity levels	11
Images per velocity level	10
Total images	110
Resolution (cropped)	900×1600 pixels
Organization	Speed-specific directories (one folder per velocity level)

**Table 3:** Computational complexity analysis of pipeline components.

Algorithm Stage	Time Complexity	Space Complexity	Dominant Operation
MAPE	$O(M\;\cdot \;N^{2})$	O(M·N)	Self-similarity computation
ROPE	O(M·N)	O(M·N)	EMA smoothing gradient
MAQ	O(M·N)	O(M·N)	Adaptive filtering
Post-processing			
Inference	$O(N_{q})$	O(1)	Polynomial evaluation
Training	O(V)	O(V)	Regression fitting
Overall	$O(M\;\cdot \;N^{2})$	O(M·N)	MAPE-limited

**Table 4:** Scalability analysis and performance characteristics.

Aspect	Analysis
Scalability	Quadratic dependency on image width (N) in MAPE limits scalability. For typical dimensions (M=400,N=400): ∼64 M operations per image.
Parallelization	Row-independent processing enables straightforward parallelization across scanlines. Effective complexity reduces to $O(N^{2})$ with *M* parallel processors.
Memory	Consistent O(M·N) footprint across all stages ensures reasonable memory requirements for practical implementations.
Optimization	Spatial downsampling or region-of-interest processing can maintain acceptable performance while preserving detection accuracy.
Bottleneck	MAPE self-similarity computation dominates overall system complexity, suggesting parallel acceleration for larger inputs.

**Figure A1:**
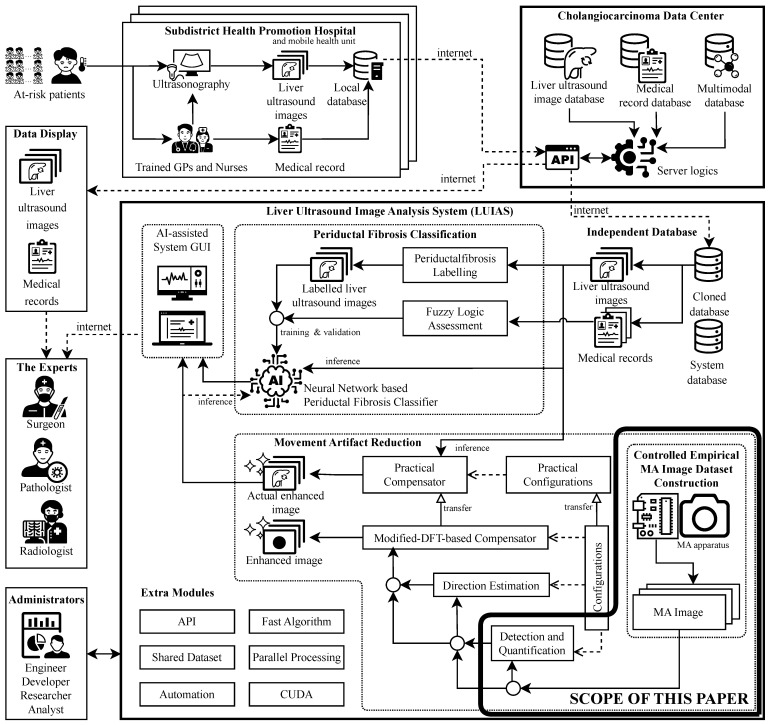
System overview of LUIAS with the coverage of this paper highlighted.

**Figure A2:**
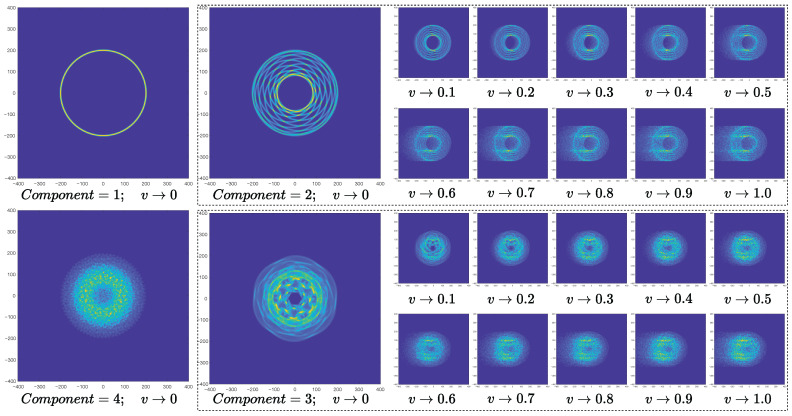
Illustrative appendix depiction of multi-component trajectory superposition. Left: zero-drift projections for increasing numbers of oscillatory components. Right: representative velocity-dependent renderings for selected multi-component cases. The shared drift term remains the one-dimensional horizontal component of the model, while the additional oscillatory components are included only to visualize how richer parametric trajectories modify the projected blur pattern. These renderings do not imply that the validated MAPE–ROPE–MAQ pipeline recovers multi-component or full three-dimensional motion from empirical images.

**Figure A3:**
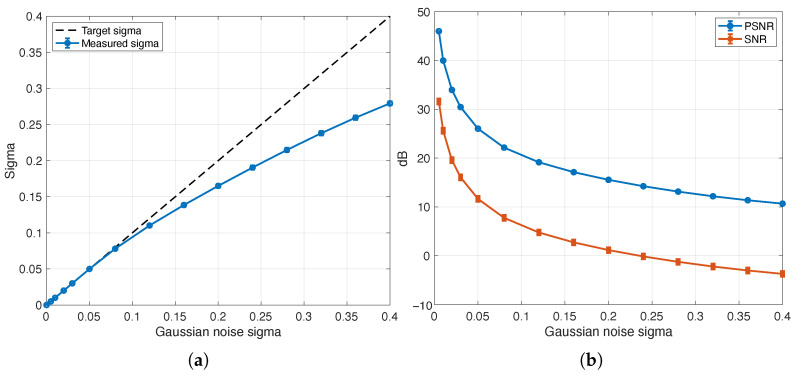
Supporting additive-noise panels retained in the appendix: (**a**) target versus measured noise strength and (**b**) image quality degradation across the perturbation ladder.

**Figure A4:**
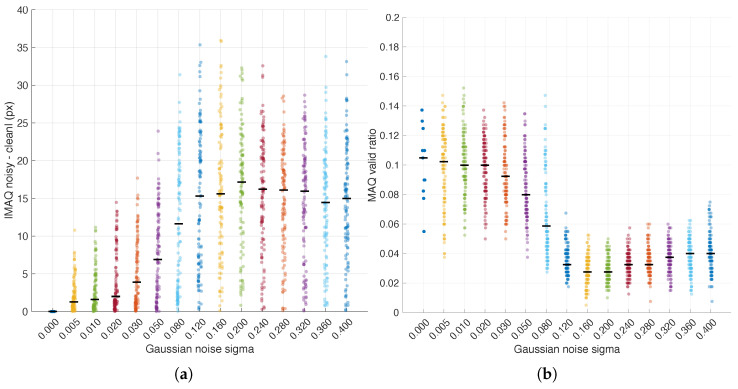
Run-level distributions of (**a**) absolute MAQ deviation from the clean baseline and (**b**) valid scanline retention ratio across noise levels.

## Data Availability

The dataset and code that support the findings of this study will be released publicly upon publication at: https://github.com/nonsakhoo/Movement-Artifact-Quantification (accessed on 19 March 2026). Until then, they are available from the corresponding author upon reasonable request.

## Abbreviations

The following abbreviations are used in this manuscript:

CASCAP	Cholangiocarcinoma Screening and Care Program
CCA	Cholangiocarcinoma
CNR	Contrast-to-Noise Ratio
CUDA	Compute Unified Device Architecture
DC	Direct Current
DFT	Discrete Fourier Transform
EMA	Exponential Moving Average
I^2^C	Inter-Integrated Circuit
ISO	ISO speed (camera sensitivity setting)
JPEG	Joint Photographic Experts Group
LCD	Liquid Crystal Display
LDR	Light Dependent Resistor
LUIAS	Liver Ultrasound Image Analysis System
MA	Movement Artifact (used here as a shorthand for motion blur)
MAE	Mean Absolute Error
MAPE	Movement Artifact Position Estimation
MAQ	Movement Artifact Quantification
MSE	Mean Squared Error
PSNR	Peak Signal-to-Noise Ratio
PWM	Pulse-Width Modulation
$R^{2}$	Coefficient of Determination
RGB	Red, Green, Blue
RMSE	Root Mean Square Error
ROPE	Reference Origin Point Estimation
SNR	Signal-to-Noise Ratio

## Appendix A. Prior Medical Imaging Motivation and System Context

This appendix documents the historical motivation and system context that initially inspired this study. The technical contribution of the manuscript is a general signal-analysis approach to motion blur quantification in single-frame images. No clinical claims are made and no human data are used; the content below is provided solely as background.

Cholangiocarcinoma (CCA), or bile duct cancer, is an aggressive malignancy with high mortality. Despite increasing global morbidity and mortality, effective early diagnosis strategies remain limited [69]. The burden of CCA is severe in the Mekong region of Southeast Asia, where incidence is among the highest worldwide [70]. Most patients are diagnosed at an advanced stage, resulting in a 5-year survival rate of less than 10%. These characteristics make CCA both a clinical and public health challenge [71,72]. CCA is strongly associated with chronic inflammation of the bile duct caused by infection with the liver fluke *Opisthorchis viverrini*, which is linked to long-standing dietary habits involving the consumption of improperly cooked freshwater fish. The disease’s long incubation period (often exceeding 10 years) allows at-risk populations to unknowingly continue high-risk behaviors during asymptomatic infection. Symptoms typically emerge only at advanced, often irreversible stages.

To address this critical situation, the Cholangiocarcinoma Screening and Care Program (CASCAP) was established as a large-scale, prospective cohort initiative in Thailand. CASCAP aims to improve early detection through systematic ultrasound-based screening of populations residing in high-risk areas, while also maintaining a longitudinal registry of confirmed CCA cases [73]. Ultrasound imaging plays a central role in this program due to its accessibility, cost-effectiveness, and suitability for large-scale deployment. However, the success of such screening efforts depends not only on coverage but also on the quality and interpretability of the acquired images.

In standard clinical practice, ultrasound examinations and diagnoses are performed by senior medical personnel, such as radiologists and surgeons, who interactively manipulate the probe and integrate multiple viewing angles before reaching a conclusion. In large-scale screening programs such as CASCAP, however, the number of high-risk individuals far exceeds the availability of specialists. To increase coverage, trained general practitioners and nurses may be instructed to acquire ultrasound images according to predefined protocols. The resulting images can be stored digitally in a centralized system for subsequent expert review and triage.

To mitigate capacity mismatches in large-scale screening, the Liver Ultrasound Image Analysis System (LUIAS) was developed as a computer-assisted screening tool. It includes a Periductal Fibrosis classification module that prioritizes high-risk cases [4]. During the development of LUIAS, it became evident that automated analysis is highly sensitive to image quality and to motion-induced distortions during acquisition. These challenges motivated measure-first strategies that estimate blur-related parameters before compensation.

The proposed framework and scope of this paper are highlighted in the context of the overall LUIAS in Figure A1.

Additional historical system-context references are cited in the Introduction; this appendix is retained to document the original motivation and deployment setting without changing the domain-agnostic technical scope of this work.

## Appendix B. Equivalence Between Real-Valued and Complex Signal Representations

In the main text, the planar motion model is written in real coordinates as

$$x\left(t\right)=\left(\begin{array}{c} A\cos(\omega t)-vt\\ A\sin(\omega t) \end{array}\right),$$

and in compact complex form as

$$z\left(t\right)=Ae^{i\omega t}-vt.$$

These two expressions are equivalent because Euler’s formula gives

$$e^{i\omega t}=\cos\left(\omega t\right)+i\sin\left(\omega t\right),$$

so that

z(t)=Acos(ωt)−vt+iAsin(ωt).

Therefore,

Re(z(t))=Acos(ωt)−vt,Im(z(t))=Asin(ωt),

and the real-coordinate vector can be recovered as

x(t)=Re(z(t))Im(z(t)).

Hence, the compact complex expression and the two-dimensional real-valued trajectory encode the same planar motion. The complex notation is convenient because the oscillatory part is represented by a single exponential term, while the real-coordinate form makes the horizontal drift and vertical oscillation explicit.

## Appendix C. 3D Trajectory to 2D Projection

Let the sampled trajectory be ${\left\{\left(x_{n},y_{n},t_{n}\right)\right\}}_{n=0}^{N-1}$, where the planar coordinates $\left(x_{n},y_{n}\right)$ correspond to the real-coordinate motion model introduced in the main text. To render this trajectory on a discrete image grid, we define an image array I[i,j] and accumulate one local kernel contribution per sample. If the rendering window has size (2N+1)×(2N+1), the projected coordinates are shifted so that the origin lies near the image center:

$$x_{n}^{\prime }=x_{n}+N+1,\;y_{n}^{\prime }=y_{n}+N+1.$$

Each sampled point contributes to the surrounding pixels through a discrete Gaussian kernel

$$K\left(m,n\right)=\exp\left(-\frac{m^{2}+n^{2}}{2\sigma^{2}}\right),\;\sigma >0,$$

evaluated on a finite neighborhood of width *K*. For a pixel (i,j) in that neighborhood, the local offsets are

$$\Delta x=i-x_{n}^{\prime },\;\Delta y=j-y_{n}^{\prime },$$

and the image is updated by

I[j,i]←I[j,i]+K(Δx,Δy).

Summing over all trajectory samples yields the rendered image

$$I\left(x,y\right)=\sum_{n=0}^{N-1}K\left(x-x_{n}^{\prime },y-y_{n}^{\prime }\right).$$

This is the discrete counterpart of the accumulation model used in the main text: the 3D trajectory is first projected onto the xy-plane, then each projected sample deposits spatial energy onto nearby pixels. The resulting image provides a smooth sub-pixel approximation of the motion path suitable for visualization.

## Appendix D. Multiple-Component Trajectory Simulation

To illustrate how the illustrative trajectory model can represent richer motion patterns, we extend the single-component construction to a superposition of oscillatory components while retaining the shared horizontal drift term. This section remains illustrative: it is included only to visualize how more complex trajectories can still produce structured projections, and it does not imply that the validated MAPE–ROPE–MAQ pipeline recovers multi-component or full three-dimensional motion from a single image.

For example, a two-component trajectory can be written as

$$x\left(t\right)=\left(\begin{array}{c} A_{1}\cos\left(\omega_{1}t+\phi_{1}\right)+A_{2}\cos\left(\omega_{2}t+\phi_{2}\right)-vt\\ A_{1}\sin\left(\omega_{1}t+\phi_{1}\right)+A_{2}\sin\left(\omega_{2}t+\phi_{2}\right) \end{array}\right),$$

where $A_{j}$, $\omega_{j}$, and $\phi_{j}$ denote the amplitude, angular frequency, and phase offset of component *j*, respectively, and *v* denotes the shared horizontal drift term. The corresponding complex form is

$$z_{2}\left(t\right)=\sum_{j=1}^{2}A_{j}e^{i(\omega_{j}t+\phi_{j})}-vt.$$

This extends naturally to an *n*-component trajectory,

$$x_{n}\left(t\right)=\left(\begin{array}{c} \sum_{j=1}^{n}A_{j}\cos\left(\omega_{j}t+\phi_{j}\right)-vt\\ \sum_{j=1}^{n}A_{j}\sin\left(\omega_{j}t+\phi_{j}\right) \end{array}\right),\;z_{n}\left(t\right)=\sum_{j=1}^{n}A_{j}e^{i(\omega_{j}t+\phi_{j})}-vt.$$

Consistent with the available simulation scripts, the illustrative renders distribute amplitudes across radial partitions, spread the component frequencies over a logarithmic range, and use phase offsets to cover the rotating plane more uniformly. After embedding the path as $\left(x_{n}\left(t\right),y_{n}\left(t\right),t\right)$, the Gaussian-kernel projection procedure described in Appendix C yields a smooth 2D motion-blur pattern.

Figure A2 shows illustrative examples of this construction. The left side compares zero-drift rendered patterns for increasing component counts, including two-, three-, and four-component cases, while the right side shows representative velocity-dependent variations for selected multi-component examples. These renderings are intended only to extend the illustrative visualization of exposure-time accumulation; multi-component recovery from empirical single-frame images remains outside the validated scope of the present pipeline.

## Appendix E. Standard Error for Normalized Impulse Response

For the row-wise normalized impulse response $r_{m}\left[l\right]$, the sampling variability at a lag *l* can be summarized by a standard-error approximation of the form

$$\mathrm{SE}\left(r_{m}\left[l\right]\right)=\sqrt{\frac{1}{N}\left(1+2\sum_{j=1}^{q}r_{m}{\left[j\right]}^{2}\right)},$$

where *N* is the scanline length and *q* is a truncation lag beyond which the self-similarity is assumed negligible. This expression is consistent with the row-wise notation used in the main text and indicates that the uncertainty at lag *l* depends on both sample size and the accumulated short-lag correlation structure.

For scanlines without meaningful repeated structure, the off-zero responses satisfy $r_{m}\left[j\right]\approx 0$ for j>0, so the approximation simplifies to

$$\mathrm{SE}\left(r_{m}\left[l\right]\right)\approx \frac{1}{\sqrt{N}}.$$

In that regime, local peaks in $r_{m}\left[l\right]$ are more plausibly attributed to random fluctuation than to stable MA structure. The standard-error perspective is therefore useful when interpreting weak or marginal second-peak detections.

## Appendix F. Angular Velocity Calculation

The relationship between angular displacement and linear velocity *v* for a rotating part is governed by:

v=r·ω

where r=0.5m is the radius of rotation, ω is the angular velocity in rad/s, derived from:

$$\omega =\frac{2\pi }{T}$$

with *T* being the period of one full rotation, which is measured by recording the time lapse Δt when the marker interrupts the laser beam:

$$T=\Delta t=\frac{\mathrm{elapse}}{1000.0}$$

Thus, the linear speed is:

$$v=\frac{\pi \;\cdot \;\mathrm{shaft}\text{\_}\mathrm{diameter}}{\Delta t}$$

The system accepts the measured velocity $v_{\mathrm{measured}}$ only if it satisfies the following stability criterion:

$$\left|v_{\mathrm{set}}-v_{\mathrm{measured}}\right|\leq \varepsilon$$

where $v_{\mathrm{set}}$ is the target velocity, ε=0.01m/s defines a 1% tolerance band. This constraint ensures velocity consistency during image acquisition, enabling accurate ground truth labeling.

## Appendix G. Contrast to Noise Ratio (CNR) Calculation

The CNR is computed to quantify the distinguishability between regions of interest and background in the preprocessed image. Given the cropped image $I\in R^{H\times W}$, two distinct regions are defined: a signal region representing the central area of potential artifact activity, and a background region comprising the remaining pixels. The signal region is defined as a rectangular mask centered within the image, with dimensions proportional to the image size:

$$w_{s}=\left\lfloor 0.7\times W\right\rfloor ,\;h_{s}=\left\lfloor 0.3\times H\right\rfloor$$

The spatial boundaries of the signal region are computed as:

$$r_{\mathrm{start}}=\left\lfloor \frac{H}{2}-\frac{h_{s}}{2}\right\rfloor ,\;r_{\mathrm{end}}=r_{\mathrm{start}}+h_{s}-1$$

$$c_{\mathrm{start}}=\left\lfloor \frac{W}{2}-\frac{w_{s}}{2}\right\rfloor ,\;c_{\mathrm{end}}=c_{\mathrm{start}}+w_{s}-1$$

This yields a binary signal mask $M_{s}\in {\left\{0,1\right\}}^{H\times W}$ where

$$M_{s}\left(i,j\right)=\left\{\begin{array}{cc} 1, & \mathrm{if}\;r_{\mathrm{start}}\leq i\leq r_{\mathrm{end}}\;\mathrm{and}\;c_{\mathrm{start}}\leq j\leq c_{\mathrm{end}}\\ 0, & \mathrm{otherwise} \end{array}\right.$$

The background mask is defined as the complement: $M_{b}=\neg M_{s}$. The pixel intensities within each region are extracted as:

$$S=\left\{I\left(i,j\right):M_{s}\left(i,j\right)=1\right\},\;B=\left\{I\left(i,j\right):M_{b}\left(i,j\right)=1\right\}$$

The statistical moments for each region are computed as:

$$\mu_{s}=\frac{1}{\left|S\right|}\sum_{p\in S}p,\;\mu_{b}=\frac{1}{\left|B\right|}\sum_{p\in B}p$$

$$\sigma_{s}^{2}=\frac{1}{|S|-1}\sum_{p\in S}{\left(p-\mu_{s}\right)}^{2},\;\sigma_{b}^{2}=\frac{1}{|B|-1}\sum_{p\in B}{\left(p-\mu_{b}\right)}^{2}$$

The contrast-to-noise ratio is then defined as:

$$\mathrm{CNR}=\frac{|\mu_{s}-\mu_{b}|}{\sqrt{0.5(\sigma_{s}^{2}+\sigma_{b}^{2})}}$$

This metric quantifies signal-to-noise ratio (CNR), indicating better contrast and lower noise interference. The denominator is the pooled standard deviation, normalizing the intensity difference relative to the combined variability of both regions. This CNR computation ensures reliable artifact detection on images with sufficient contrast.

## Appendix H. Camera Settings and the Effects of Each Variable

The camera exposure process is governed by three primary parameters: aperture (*A*), shutter speed (*S*), and ISO sensitivity (ISO). These variables influence both light integration and susceptibility to motion blur:

### Appendix H.1. Aperture (A)

Expressed as an *f*-number, aperture controls both light intake and depth of field. Mathematically, aperture diameter *D* relates to the focal length *f* as:

$$A=\frac{f}{D}$$

A smaller *A* (e.g., f/1.8) implies a larger aperture, greater light intake, and reduced depth of field.

### Appendix H.2. Shutter Speed (S)

Represents the exposure duration $\Delta t_{s}$. A lower *S* (faster shutter) reduces motion blur, defined by:

$$\mathrm{Motion}\;\mathrm{Blur}\;\mathrm{Length}=v\;\cdot \;\Delta t_{s}$$

where *v* is the object speed relative to the sensor. Hence, increasing $\Delta t_{s}$ induces more MA.

### Appendix H.3. ISO Sensitivity (ISO)

Impacts the amplification of incoming signals. Higher ISO improves brightness under low light but increases noise variance $\sigma_{n}^{2}$, following:

$$\sigma_{n}^{2}\propto \mathrm{ISO}$$

For all image acquisitions, the camera settings were configured as follows: aperture f/1.8, shutter speed 1/60, ISO set to auto (approximately 560), white balance at 4000 K, and manual focus mode. These parameters were held constant to promote consistent exposure and reduce confounding factors in MA observation; under this controlled protocol, variations in MA are interpreted as primarily driven by object speed *v* rather than changes in optics or electronics.

## Appendix I. Movement Artifact Generator System Architecture

The movement artifact generator system produces controlled movement artifacts in imaging scenarios to evaluate MA detection algorithms. It integrates hardware and software to precisely control rotational speed and image capture timing.

### Appendix I.1. Hardware Configuration

The movement artifact generator employs an Arduino-based microcontroller system with integrated sensor and actuator modules. The hardware architecture consists of a Light Dependent Resistor (LDR) connected to interrupt pin 3 for shaft rotation detection, a laser diode on pin 7 for illumination, and camera control interfaces utilizing pins 8 and 9 for focus and capture operations, respectively. A push-button interface on interrupt pin 2 enables manual system triggering, while a 16 × 4 I^2^C LCD display (address 0x3F) provides real-time parameter visualization.

### Appendix I.2. Speed Measurement Algorithm

The system implements a precision timing mechanism for rotational speed calculation based on shaft geometry. With a predefined shaft diameter of 0.5 m and 20 equally-spaced holes, the circumferential distance per hole is calculated as π×diameter. Speed determination utilizes interrupt-driven timing measurements, where consecutive LDR triggers establish elapsed time intervals. The speed calculation follows the relationship: $\mathrm{speed}=\frac{\mathrm{circumference}}{\mathrm{elapsed}\text{\_}\mathrm{time}}$, providing real-time velocity feedback in meters per second.

### Appendix I.3. Control Logic Implementation

During initialization phase, system parameters including shaft diameter (0.5 m), camera distance (1.0 m), and speed error tolerance (0.01 m/s) are displayed for 10 s or until manual activation. The operational phase implements a multi-threaded approach using interrupt service routines for LDR detection and button press events, ensuring microsecond-precision timing accuracy.

### Appendix I.4. Camera Synchronization Protocol

Precise image capture synchronization employs a three-condition trigger mechanism: button activation, camera focus confirmation, and speed stability verification. Speed stability is achieved when the absolute difference between target and measured velocities remains within the configured tolerance (±0.01 m/s). Upon meeting all conditions, the system executes a programmable delay before camera activation, accommodating object positioning requirements.

### Appendix I.5. Serial Communication Interface

The system uses a CSV-based serial protocol for real-time parameter adjustment. Input parameters include capture delay (milliseconds), voltage reference, pulse rate (pulses per second), and target speed (m/s). This interface allows dynamic system reconfiguration without code changes, supporting experimental flexibility and automated control integration.

## Appendix J. Supplementary Details for the Additive-Noise Robustness Analysis

This appendix provides the formal metric definitions and supplementary figures for the extended additive-noise robustness study summarized in Section 4.11. Zero-mean Gaussian noise was added to each cropped image at normalized standard deviations

$$\begin{array}{cc} \sigma_{n}\in & \{0.000,0.005,0.010,0.020,0.030,0.050,0.080,\\ \; & 0.120,0.160,0.200,0.240,0.280,0.320,0.360,0.400\} \end{array}$$

and each level was repeated 10 times with fixed random seeds across 11 images, yielding 1650 noisy realizations. The perturbation ladder was intentionally extended until the marker became barely observable at the highest levels, so these results should be interpreted as boundary characterization rather than as a nominal operating benchmark.

Let $C_{k}\in {\left[0,1\right]}^{H\times W}$ denote the *k*-th cropped image after grayscale standardization and contrast preparation, indexed over the selected image subset k=1,…,K with K=11. For target noise level $\sigma_{n}$ and Monte Carlo repeat r=1,…,R with R=10, the noisy realization supplied to the preprocessing and MAPE–ROPE–MAQ pipeline is modeled as

(A1) $$Y_{k,r}^{\left(\sigma_{n}\right)}\left(x,y\right)=\Pi_{\left[0,1\right]}\;\left(C_{k}\left(x,y\right)+\sigma_{n}\;\varepsilon_{k,r}\left(x,y\right)\right),\;\varepsilon_{k,r}\left(x,y\right)\overset{i.i.d.}{∼}N\left(0,1\right),$$

where $\Pi_{\left[0,1\right]}\left(\cdot \right)$ denotes clipping to the unit intensity interval. The realized perturbation field is then

(A2) $$E_{k,r}^{\left(\sigma_{n}\right)}\left(x,y\right)=Y_{k,r}^{\left(\sigma_{n}\right)}\left(x,y\right)-C_{k}\left(x,y\right).$$

To distinguish the intended noise level from the effective perturbation after clipping, we summarize each realization by the measured perturbation standard deviation, mean squared error, peak signal-to-noise ratio, and signal-to-noise ratio:

(A3) $$\begin{array}{cc} {\hat{\sigma }}_{k,r}^{\left(\sigma_{n}\right)} & =\sqrt{\frac{1}{HW-1}\sum_{x=1}^{W}\sum_{y=1}^{H}{\left(E_{k,r}^{\left(\sigma_{n}\right)}\left(x,y\right)-{\bar{E}}_{k,r}^{\left(\sigma_{n}\right)}\right)}^{2}}, \end{array}$$

(A4) $$\begin{array}{cc} {\mathrm{MSE}}_{k,r}^{\left(\sigma_{n}\right)} & =\frac{1}{HW}\sum_{x=1}^{W}\sum_{y=1}^{H}{\left(E_{k,r}^{\left(\sigma_{n}\right)}\left(x,y\right)\right)}^{2}, \end{array}$$

(A5) $$\begin{array}{cc} {\mathrm{PSNR}}_{k,r}^{\left(\sigma_{n}\right)} & =10\log_{10}\;\left(\frac{1}{{\mathrm{MSE}}_{k,r}^{\left(\sigma_{n}\right)}}\right), \end{array}$$

(A6) $$\begin{array}{cc} {\mathrm{SNR}}_{k,r}^{\left(\sigma_{n}\right)} & =10\log_{10}\;\left(\frac{\frac{1}{HW}\sum_{x=1}^{W}\sum_{y=1}^{H}C_{k}{\left(x,y\right)}^{2}}{{\mathrm{MSE}}_{k,r}^{\left(\sigma_{n}\right)}}\right), \end{array}$$

where ${\bar{E}}_{k,r}^{\left(\sigma_{n}\right)}$ is the spatial mean of the perturbation field, the PSNR expression uses the unit peak intensity of the normalized images, and the SNR expression measures the ratio between clean-image power and perturbation power.

Figure A3 provides the first layer of appendix support for these definitions by showing how closely the realized perturbation strength follows the target noise ladder after clipping and how image quality degrades across the same perturbation range. These panels establish the noise-characterization context before turning to stage-wise retention and MAQ-response behavior.

For each processing stage S∈{MAPE,ROPE,MAQ}, let $u_{k,r,m}^{\left(S,\sigma_{n}\right)}$ denote the row-wise output on scanline m=1,…,M. The mean valid ratio reported for stage *S* at noise level $\sigma_{n}$ is defined by

(A7) $$\eta_{S}\left(\sigma_{n}\right)=\frac{1}{KRM}\sum_{k=1}^{K}\sum_{r=1}^{R}\sum_{m=1}^{M}1\;\left\{u_{k,r,m}^{\left(S,\sigma_{n}\right)}\;\mathrm{is}\;\mathrm{finite}\right\},$$

so that $\eta_{S}\left(\sigma_{n}\right)\in \left[0,1\right]$ measures the fraction of retained scanline-level detections or pairings after filtering.

To summarize the image-level MAQ response, let $q_{k,r,m}^{\left(\sigma_{n}\right)}$ denote the row-wise MAQ displacement on scanline *m* and let

(A8) $$V_{k,r}^{\left(\sigma_{n}\right)}=\left\{m\in \left\{1,\ldots ,M\right\}:q_{k,r,m}^{\left(\sigma_{n}\right)}\;\mathrm{is}\;\mathrm{finite}\right\}$$

be the set of scanlines that survive the MAQ plausibility filtering. The image-level MAQ estimate and its absolute deviation from the clean baseline are then written as

(A9) $$\begin{array}{cc} Q_{k,r}^{\left(\sigma_{n}\right)} & =\left\{\begin{array}{cc} \frac{1}{\left|V_{k,r}^{\left(\sigma_{n}\right)}\right|}\sum_{m\in V_{k,r}^{\left(\sigma_{n}\right)}}q_{k,r,m}^{\left(\sigma_{n}\right)}, & \left|V_{k,r}^{\left(\sigma_{n}\right)}\right|>0,\\ \mathrm{NaN}, & \left|V_{k,r}^{\left(\sigma_{n}\right)}\right|=0, \end{array}\right. \end{array}$$

(A10) $$\begin{array}{cc} \Delta_{k,r}^{\left(\sigma_{n}\right)} & =\left|Q_{k,r}^{\left(\sigma_{n}\right)}-Q_{k}^{\left(0\right)}\right|, \end{array}$$

where $Q_{k}^{\left(0\right)}$ is the clean-image MAQ estimate for the same cropped image. The appendix reports the mean absolute MAQ drift

(A11) $$\bar{\Delta }\left(\sigma_{n}\right)=\frac{1}{KR}\sum_{k=1}^{K}\sum_{r=1}^{R}\Delta_{k,r}^{\left(\sigma_{n}\right)},$$

which directly measures how far the noisy MAQ estimate moves away from the clean baseline at the same image content.

To summarize outright image-level breakdown, we define the failure indicator

(A12) $$\chi_{k,r}^{\left(\sigma_{n}\right)}=1\;\left\{Q_{k,r}^{\left(\sigma_{n}\right)}\;\mathrm{is}\;\mathrm{not}\;\mathrm{finite}\right\},$$

and the corresponding failure rate

(A13) $$f\left(\sigma_{n}\right)=\frac{1}{KR}\sum_{k=1}^{K}\sum_{r=1}^{R}\chi_{k,r}^{\left(\sigma_{n}\right)}.$$

Figure A4 then summarizes the run-level dispersion of the two quantities most directly tied to downstream robustness interpretation: absolute MAQ deviation from the clean baseline and valid scanline retention across repeated noisy realizations. Presenting these distributions here makes the spread behind the trend-level main-text discussion explicit.

Across the selected subset, the clean baseline produced a mean MAQ of 32.36 pixels with mean valid ratios of 0.854 for MAPE, 0.972 for ROPE, and 0.101 for MAQ. At an intermediate perturbation level ($\sigma_{n}=0.120$), the mean absolute MAQ deviation from the clean baseline was 14.97 pixels, the mean MAQ valid ratio was 0.036, and the mean PSNR was 19.15 dB. At the highest tested perturbation ($\sigma_{n}=0.400$), where the target became barely observable, the mean absolute MAQ deviation was 14.45 pixels, the mean MAPE, ROPE, and MAQ valid ratios were 1.000, 0.695, and 0.041, respectively, the mean PSNR was 10.67 dB, and the recorded failure rate remained 0.000. These numerical milestones are consistent with the trend-level interpretation emphasized in the main text: candidate self-similarity responses can survive heavy perturbation, but the downstream stages lose stable support more quickly.

Taken together, Figure A3 and Figure A4 show that the perturbation ladder remains well-characterized while the downstream response becomes increasingly dispersed and selectively retained at heavier noise levels. These supplementary measurements indicate that strong additive interference can still produce candidate scanline self-similarity responses, but downstream reference-origin localization and MAQ pairing become markedly less reliable under severe perturbation. In that sense, the appendix complements the main-text robustness subsection by documenting how measured noise strength, image quality degradation, and run-level dispersion evolve across the full perturbation ladder. The results should not be interpreted as proof of robustness to arbitrary textured backgrounds, lower-contrast targets, or uncontrolled natural scenes.
